# Directing Ion Transport and Interfacial Chemistry in Pnictogen-Substituted Thio-LISICONs

**DOI:** 10.1021/acsami.4c22390

**Published:** 2025-03-20

**Authors:** Philip Yox, Glenn Teeter, Lucas Baker, Drew Whitney, Annalise E. Maughan

**Affiliations:** † Department of Chemistry, Colorado School of Mines, Golden, Colorado 80401, United States; ‡ National Renewable Energy Laboratory, Golden, Colorado 80401, United States

**Keywords:** thio-LISICON, solid electrolyte, aliovalent substitution, interfacial stability, X-ray photoelectron spectroscopy

## Abstract

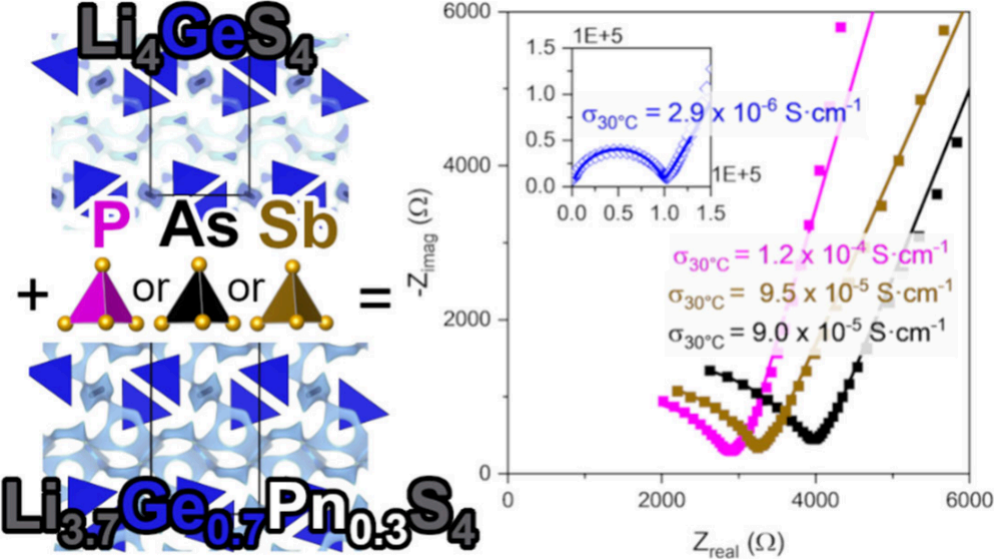

Aliovalent substitution is a ubiquitous strategy to increase ionic conductivity in solid-state electrolytes, often by many orders of magnitude. However, the structural and compositional changes that occur upon aliovalent substitution are highly interrelated, and a deep understanding of how substitutions simultaneously impact ion transport and the chemical evolution of interfaces during electrochemical cycling remain as prevailing challenges. Here, we interrogate aliovalent pnictogen substitution of Li_4_GeS_4_ in the series Li_3.7_Ge_0.7_Pn_0.3_S_4_ (Pn = P, As, Sb) and unravel the impact on ion transport processes and degradation during electrochemical cycling. High-resolution powder X-ray diffraction and pair distribution function analysis reveal that all substituted compounds exhibit an anisometric distortion of the Li_4_GeS_4_ structure. Temperature-dependent potentiostatic electrochemical impedance spectroscopy reveals that aliovalent substitution increases the room-temperature lithium ionic conductivity by 2 orders of magnitude. Curiously, aliovalent substitution results in a simultaneous increase in the Arrhenius prefactor and decrease in the activation barrier, which contribute to the significant increase in lithium-ion conductivity. We attribute this apparent violation of the “Meyer–Neldel” entropy-enthalpy compensation to the introduction of Li^+^ vacancies that elicit a redistribution of the lithium substructure. Electrochemical stability and cycling performance were interrogated by critical current density tests on symmetric cells with Li electrodes coupled with virtual electrode X-ray photoelectron spectroscopy measurements. In all substituted compounds, we observe the growth of electronically conductive phases that result in continual growth of the solid electrolyte interphase and increase in interfacial impedance during electrochemical cycling. We find that electrochemical instability against Li^0^ is predominantly driven by reduced Ge species. Taken together, our study presents holistic insights into the structural and compositional factors that drive ionic conductivity and electrochemical degradation in lithium metal sulfide solid-state electrolytes.

## 1 Introduction

Better batteries are expected to provide storage solutions to intermittent renewable energy generation and transportation.^1^ Solid-state batteries present an opportunity to remarkably enhance energy densities as well as tackle challenges such as fast charging.^2^ While solid electrolytes could enable these new battery features, they pose substantial challenges for material selection. Solid electrolytes must exhibit high ionic conductivity, be electrochemically stable to the cathode and anode, conform well to electrode interfaces, and suppress dendrite formation.^3^

Solid electrolyte materials must have excellent ionic conductivity, preferably higher than liquid electrolytes (∼10 mS/cm). Aliovalent substitution is a common strategy to improve ionic conductivity in solid-state electrolytes; the substitution of differently charged species in the host framework is compensated by changes in the Li^+^ concentration. This strategy has been widely employed and while the desired effect (higher ionic conductivity) is often achieved, the role of the substituted element is not always understood or at least the consequence of the substitution is convoluted. The argyrodite structure type has had a myriad of studies showing aliovalent substitution increases ion conductivity. In one case the compound Li_6+x_P_1–*x*_Ge_*x*_S_5_I claims to induce anion mixing through the P/Ge substitution, which realizes high ionic conductivity.^4^ However, another study of a very similar material Li_6+x_Sb_1–*x*_Ge_*x*_S_5_I shows no indication of anion mixing, yet the ionic conductivity reaches similar levels as the previous material.^5^ It is possible that P/Ge and Sb/Ge substitutions are fundamentally different but result in the same outcome. Regardless, determining the role of the substituted atom is critical for future development of solid electrolytes through structural and compositional design principles. Choosing the best candidate for substitution may decrease the amount of trial-and-error in solid electrolyte ionic conductivity enhancement. In regard to the other important qualities a solid electrolyte must attain, substitutions and even composite doping of solid electrolytes have claimed to modify electrochemical stability of interfacial reactions.^6−8^

The thio-LISICONs (Li SuperIonic CONductors) are a class of materials with the general formula Li_8-x_M^x+^S_4_ (M = main group metal: Al, Ga, Si, Ge, Sn, P, As, Sb).^9^ The structure of thio-LISICONs contains isolated MS_4_ polyhedra, similar to the argyrodites, theoretically allowing 3-dimensional Li diffusion pathways. Unlike the argyrodites, the ternary thio-LISICONs do not have any anion disorder simply due to S being the only anion. Thus, substitution of main group metals can only influence disorder on the metal and Li sublattices. These materials adopt a relatively simple structure and are amenable to substitutions with a large range of main group metals. This work serves to probe the unresolved and underlying correlations between the choice of elements for an aliovalent substitution and the structure and ion transport properties. For this study we investigated the substitution of P, As, and Sb on Li_4_GeS_4_. We seek to holistically understand the role of the substituted atom on ionic conductivity, ion conduction pathways and migration energies, as well as electrochemical stability.

## 2 Experimental Methods

### 2.1 Synthesis

All materials were synthesized via a two-step process involving a ball-mill step and subsequent annealing. Additionally, due to the air-sensitive nature of both products and reactants, all sample manipulations were carried out in an Ar glovebox with <0.2 ppm of O_2_ and H_2_O. Li_2_S powder, Ge powder, the appropriate pnictogen metal powder (P, As, Sb), and sulfur powder were combined and mixed thoroughly in a mortar and pestle (2.0 g scale) before being transferred to a zirconia ball mill jar with twenty-five 10 mm zirconia media. The zirconia jar was sealed inside of a stainless-steel sheath which was hermetically sealed before removal from the glovebox. The milling parameters were optimized individually for each composition (Table 1). 250 mg of the ball-milled reactants were then loaded into a fused silica ampule which had been carbonized. The ampule was flame-sealed and annealed for 12 h in a muffle furnace. The annealing temperature was also optimized individually for each composition (Table 1).

**Table 1:** Synthetic Parameters of Ball-Milling and Annealing Steps Used for the Materials

Composition	Ball Mill speed (rpm)	Total Time (h)	On/Off (min)	Annealing Temp (°C)
Li_4_GeS_4_	800	30	30/5	700
Li_3.7_Ge_0.7_P_0.3_S_4_	400	15	15/5	700
Li_3.7_Ge_0.7_As_0.3_S_4_	800	30	15/5	400
Li_3.7_Ge_0.7_Sb_0.3_S_4_	400	15	15/5	550

### 2.2 X-ray Diffraction and Total Scattering

Following synthesis as described above, samples were loaded in quartz capillaries (ID = 0.7 mm, OD = 0.72 mm) and sealed with a two-part epoxy. For high-resolution powder X-ray diffraction, all materials were diluted to 50 wt % with finely ground amorphous silica. Samples for total scattering were not diluted. Data collection for high-resolution powder X-ray diffraction (HR-PXRD) and total scattering was performed at the Canadian Light Source (CLS) BXDS-WLE (λ = 0.81931 Å) and BXDS-WHE (λ = 0.20750 Å), respectively. HR-PXRD were analyzed in TOPAS using the Rietveld method. Total scattering data was integrated into *Q*-space using GSAS-II after applying an appropriate mask and polarization corrections.^10^ The normalized total scattering patterns *S*(*Q*) were produced by subtracting background scattering from an empty quartz capillary, utilizing the appropriate sample compositions, and applying standard corrections for the area detector setup. Pair distribution function patterns for Li_4_GeS_4_, Li_3.7_Ge_0.7_P_0.3_S_4_, and Li_3.7_Ge_0.7_As_0.3_S_4_ were calculated via Fourier transformation of the total scattering data utilizing *Q*_max_ = 24 Å^–1^, while Li_3.7_Ge_0.7_Sb_0.3_S_4_ utilized *Q*_max_ = 22 Å^–1^ and smoothed with Lorch damping to suppress Fourier termination ripples. Pair distribution functions were fit with PDFgui.^11^

### 2.3 Electrochemical Impedance Spectroscopy

Potentiostatic electrochemical impedance spectroscopy (PEIS) was used to probe the ionic conductivity and activation energy of bulk lithium ion transport. The solid electrolyte powders were uniaxially pressed into pellets (O.D. = 6 mm) with a stainless steel die in a hydraulic press at ∼790 MPa inside a glovebox. The pellet was placed in a custom-built polyether ether ketone (PEEK) cell. The pellets were contacted on either side with graphite electrodes and 6 mm stainless steel rods were employed to provide electrical contact and pressure to the pellets. The cell was placed in a spring jig to maintain a constant pressure of 40 MPa with an in-line load cell to monitor pressure. PEIS data were collected on a Gamry Interface 1010E potentiostat. The frequency was swept from 2 MHz to 0.2 Hz with an applied voltage bias of 20 mV. For temperature-dependent studies, the pressure jig and cell were placed in a convection oven (Quincy Lab 10GCE) and the oven was allowed to dwell at each temperature (30 °C, and 10 °C increments between 35–95 °C) for 1.5 h after equilibrating, with spectra collected continuously with a 5 min wait step between each data collection.

### 2.4 Critical Current Density

Critical current density experiments were conducted to determine the critical current density of the materials as well as to probe the electrochemical stability of the materials against Li metal. The solid electrolyte powders were densified in the custom-built PEEK cells at a pressure of ∼40 bar. Five mm Li metal foils (MTI, 99.9%) were placed on each side of the pellet as electrodes. Stainless steel rods were used to contact the Li|SSE|Li stack and the PEEK cell was placed in the pressure jig (described above) to apply a pressure of 25 MPa for 30 min. The pressure was then reduced to 5 MPa for the duration of the critical current density experiment. The experiment was conducted with a Gamry Interface 1010E potentiostat. A program consisting of 10 min galvanostatic holds followed by PEIS was repeated with alternating current directions until either the cell became too resistive or the cell shorted. The galvanostatic holds started with a current density of 10 μA/cm^2^ and increased by 10 μA/cm^2^ every other galvanostatic hold.

### 2.5 Virtual Electrode X-ray Photoelectron Spectroscopy

VE-XPS measurements were performed with a Physical Electronics Phi VersaProbe III instrument using monochromatic Al-kα X-rays (hν = 1486.7 eV). XPS spectra were collected with a 69 eV pass energy. During the VE-XPS experiments, electron bias current was supplied via a low-energy (10 eV) electron flood gun that was turned on and off for alternating sets of high-resolution XPS core-level spectra. The electron flux impinging on the surface creates a net negative surface charge density that drives Li^+^ ion migration to the surface. At the surface, Li^+^ ions and excess electrons react with SSE compounds to form SEI phases. The appearance of metallic Li^0^ in these experiments typically signifies a degree of SEI passivation with respect to electronic conduction.

## 3 Results and Discussion

### 3.1 Synthesis

The compound Li_4_GeS_4_ as well as the aliovalent substitutions Li_3.7_Ge_0.7_Pn_0.3_S_4_ (Pn = P, As, Sb) were synthesized through a two-step procedure. First, the reactants Li_2_S, Ge, the appropriate pnictogen metal powder, and sulfur were mixed in the appropriate stoichiometric amounts corresponding to the balanced chemical equations (eqs 1 and 2). The powders were ball-milled for 15−30 h at 400–800 rpm depending on the composition. Subsequently, the ball-milled powders were annealed to obtain high-purity powders of the target phases (see Experimental Methods, Table1 for full details). After ball-milling, all samples exhibited a black color due to the presence of Ge metal. Laboratory powder X-ray diffraction (PXRD) of the ball-milled materials revealed that short times and slow rotation speeds simply led to the reduction of particle size with the crystalline phases corresponding to the precursors observed. However, longer times and faster rotation speeds led to the absence of diffraction peaks from Li_2_S, S, and the pnictogen metal precursors, presumably due to amorphization. The annealed Li_4_GeS_4_ is a white powder while the substituted compounds exhibited a beige color.
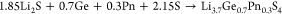
1
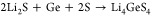
2

### 3.2 Crystal Structure

**Figure 1:**
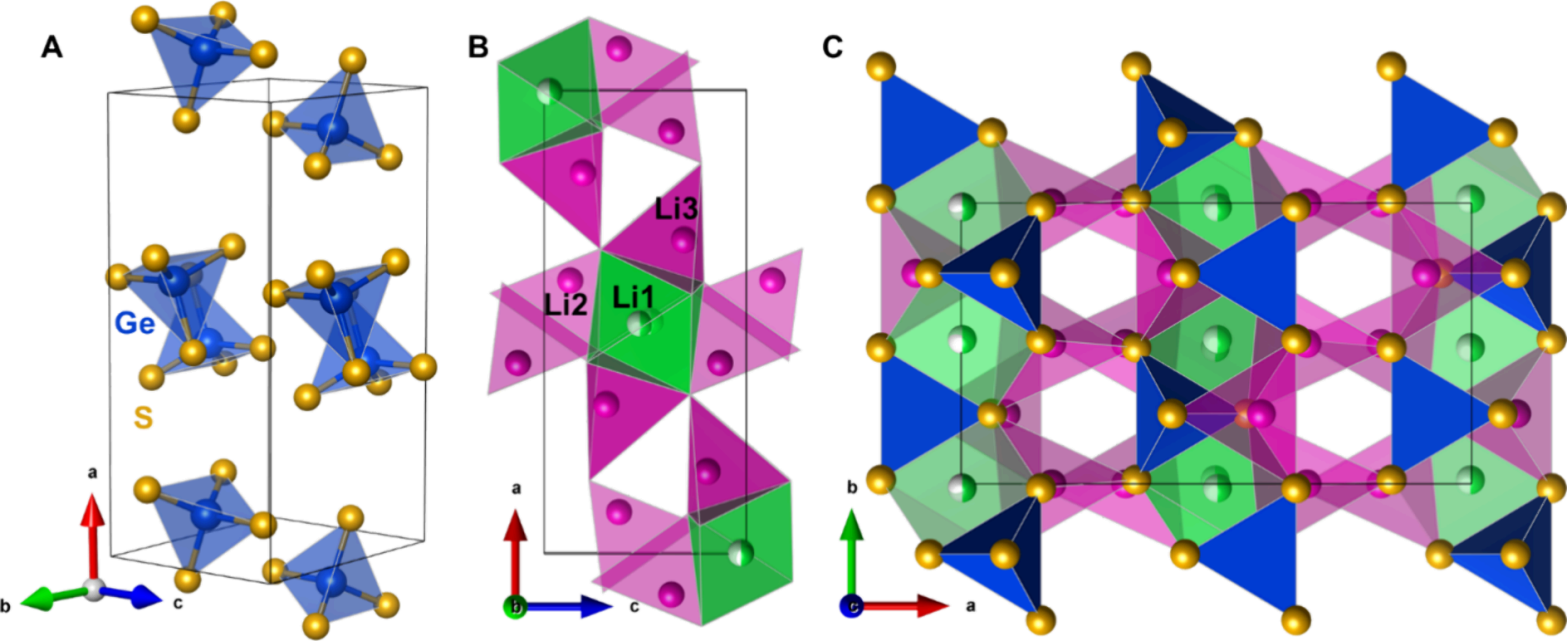
(A) A unit cell of Li_4_GeS_4_ showing the isolated GeS_4_ tetrahedra (blue). (B) The 3 unique Li positions with octahedral Li1 shown in green and tetrahedral Li2 and Li3 shown in pink. (C) A different view of the crystal structure showing the connectivity of the Li (pink and green) and Ge (blue) polyhedra

**Figure 2:**
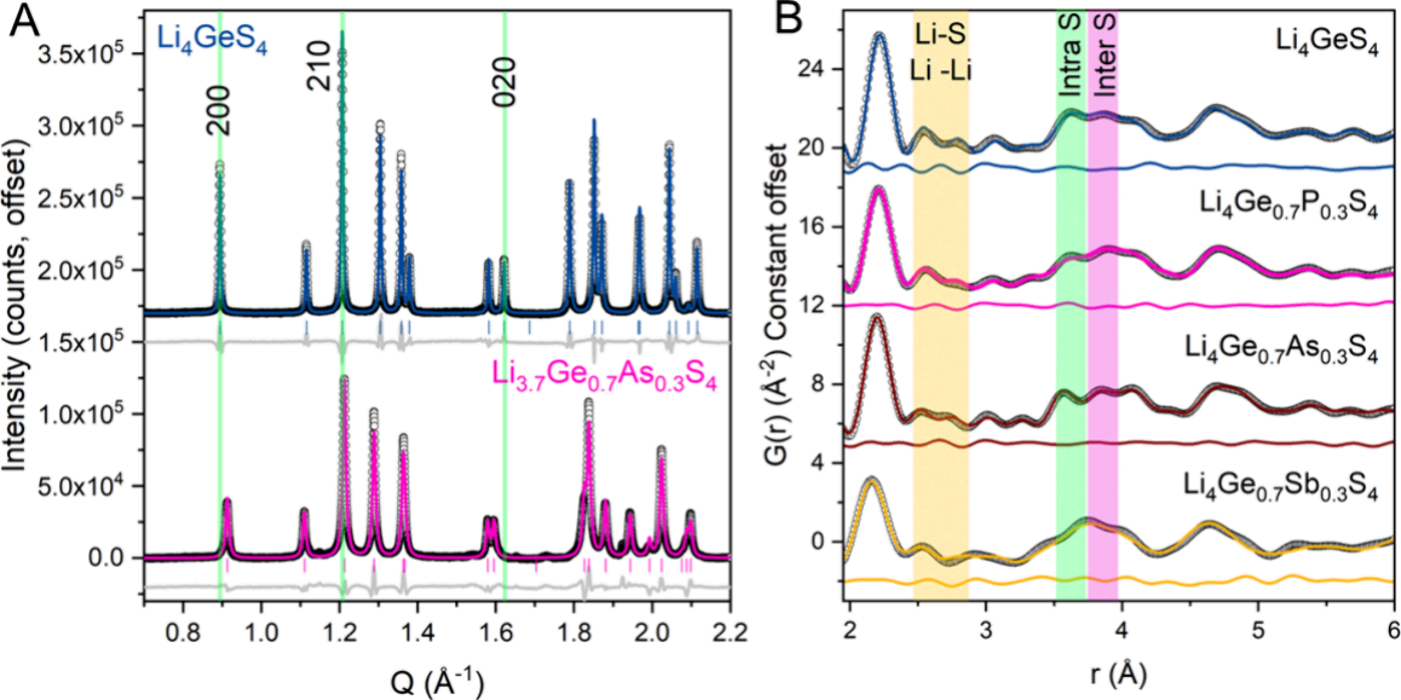
(A) Comparison of high-resolution powder X-ray diffraction of Li_4_GeS_4_ (blue) and Li_3.7_Ge_0.7_As_0.3_S_4_ (pink). Li_3.7_Ge_0.7_As_0.3_S_4_ exhibits an anisometric change in the unit cell as evidenced by the 200 and 020 reflections shifting in opposite directions relative to Li_4_GeS_4_. (B) The X-ray pair distribution function of Li_4_GeS_4_ (blue) and Li_3.7_Ge_0.7_Pn_0.3_S_4_ (Pn = P, As, Sb) are compared to show potential difference in the local order of the materials. A subtle contraction of the intratetrahedral S–S pair (green region) distance can be seen for P, As. The inter tetrahedral S–S pair distance (pink region) remains relatively unchanged. A redistribution of intensities in the Li–S and Li–Li pairs (yellow region) is mostly obscured by the low X-ray scattering of Li and termination ripples.

The structure of Li_4_GeS_4_ is composed of isolated [GeS_4_]^4–^ tetrahedra with Li^+^ ions occupying both tetrahedral and octahedral holes formed by the sulfur atoms of the GeS_4_ tetrahedra (Figure 1A-B). Li_4_GeS_4_ has 3 different Li positions as originally determined by Matsushita and Kanatzidis.^12^ The Li1 site occupies the octahedron and has been refined as an off-center split position^12^ as well as fully occupied in the center of the octahedron with a rather large anisotropic displacement parameter.^13^ Li2 and Li3 occupy tetrahedral sites with Wyckoff positions 4c and 8d, respectively. Li2 and Li3 as well as Li2 and Li1 are corner-sharing, while Li1 and Li3 are face-sharing along the *a*-*b* plane (Figure 1B–C). High-resolution powder X-ray diffraction (HR-PXRD) and total scattering experiments were performed at the Canadian Light Source (BXDS-WLE, BXDS-WHE) and subsequently Rietveld refinements and pair distribution function analysis was performed to obtain lattice parameters and atomic positions of the Li_4_GeS_4_ and substituted materials (Figure 2). HR-PXRD revealed Li_4_GeS_4_ and Li_3.7_Ge_0.7_As_0.3_S_4_ to be phase pure, with no unindexed peaks. However, Li_3.7_Ge_0.7_P_0.3_S_4_ exhibits some low intensity peaks that cannot be indexed in the *Pnma* unit cell and are attributed to a minor impurity phase. The Ge/P occupancy in Li_3.7_Ge_0.7_P_0.3_S_4_ is highly correlated with the isotropic atomic displacement parameter of the Ge/P position but fits well when fixed to the nominal composition (Ge occupancy = 0.7). The sample with nominal composition Li_3.7_Ge_0.7_Sb_0.3_S_4_ contains 10 wt % of Li_17_Sb_13_S_18_. However, refining the occupancy of the Ge/Sb site resulted in a robust Ge occupancy of 0.721 ± 0.006. To explain the seeming Sb excess of the sample we hypothesize that the impurity phase Li_17_Sb_13_S_18_ is off-stoichiometric and possibly contains some Ge. Despite the minor impurity phases present in the sample, the subsequent properties of the samples (vide infra) are dominated by the target compositions of Li_3.7_Ge_0.7_Pn_0.3_S_4_ (Pn = P, As, Sb).

In all the pnictogen substituted materials, we observe anisometric changes to the unit cell upon substitution of the pnictogen. Anisometric expansion/contraction are common in aliovalently substituted thio-LISICONs and are thought to be indicative of the changing Li^+^ distribution within the lattice.^14,15^ The volume of the unit cell remains constant with P as the substituting ion, but expands for As and Sb substitutions (Table S1). Compared to pristine Li_4_GeS_4_ the *a* lattice parameter shrinks, and the *b* lattice parameter expands when the Ge site is substituted with a pnictogen. This is clearly evidenced by the shift to higher *Q* of the 200 reflection while the 020 shifts to lower *Q* in Li_3.7_Ge_0.7_As_0.3_S_4_ (Figure 2a). At the 30% substitution level for all pnictogens the *a*/*b* ratio is nearly the same at 1.76(1) which is substantially smaller than the undoped *a*/*b* ratio of 1.812(1) in pristine Li_4_GeS_4_. Since the *a*/*b* ratio does not correlate with atomic radii of the substituted pnictogen nor unit cell volume, the *a*/*b* ratio could be an indicator of Li concentration or a redistribution of Li among the voids within the structure.^15,14,16^ While the Li occupation cannot be reliably determined from our X-ray diffraction experiments, the Li polyhedral volume is dictated by the position of the neighboring sulfur atoms and can readily be determined from X-ray scattering. Across the substituted materials we observe an enlargement of Li1 and Li2 polyhedral volumes while Li3 decreases in size (Table S3). An enlarged polyhedral volume has been corroborated by neutron diffraction to correlate with a decrease in Li occupation.^14^ Our results indicate the occupation of Li1 and Li2 are decreasing relative to Li3 with aliovalent substitution.

The average structure determined from HR-PXRD clearly shows anisometric lattice changes that may influence ion conduction. We also probed the local structure, as it is also known to heavily influence ionic conductivity.^17−19^ Additionally, the synthesis method of ball-milling is known to produce amorphous products which would not be observed in diffraction but could be observed using total scattering techniques.^20,21^ X-ray total scattering experiments were conducted, and pair-distribution function (PDF) analysis was performed to probe the local structure of Li_4_GeS_4_ and Li_3.7_Ge_0.7_Pn_0.3_S_4_ (Pn = P, As, Sb). The local structure of Li_4_GeS_4_ (1–10 Å) does not substantially deviate from the average structure refined against the HR-PXRD and all features in the PDF are accounted for. Likewise, the local structure of the substituted compounds reasonably agrees with the respective average structures and lattice parameters determined from HR-PXRD. The effect of the pnictogen substitution on the local structure is quite small; the average Ge/Pn–S distance remains constant near 2.22 Å suggesting insufficient resolution to deconvolute any contributions from distinct Pn-S or Ge–S pairs. A small contraction in the S–S distance corresponding with the intratetrahedral S–S pair (Figure 2B) is observed for Li_3.7_Ge_0.7_P_0.3_S_4_ and Li_3.7_Ge_0.7_As_0.3_S_4_ and is consistent with the substitution of a central atom (P^5+^, As^5+^) with a smaller ionic radius. The intertetrahedral S–S pairs are of great interest, as they form the diffusion bottlenecks for Li atoms to pass through. There are subtle changes on the S–S intertetrahedral distances with cation substitution observed in Rietveld or PDF (Table S5) with an average increase in the S–S distances of 0.05–0.15 Å. While our structural analysis confirms that pnictogen substitution modifies the original Li_4_GeS_4_ lattice, the small deviations in the local structure and bonding means that differences in properties are predominantly driven by changes in the Li concentration.

### 3.3 Ion Conduction and Activation Energy

**Figure 3:**
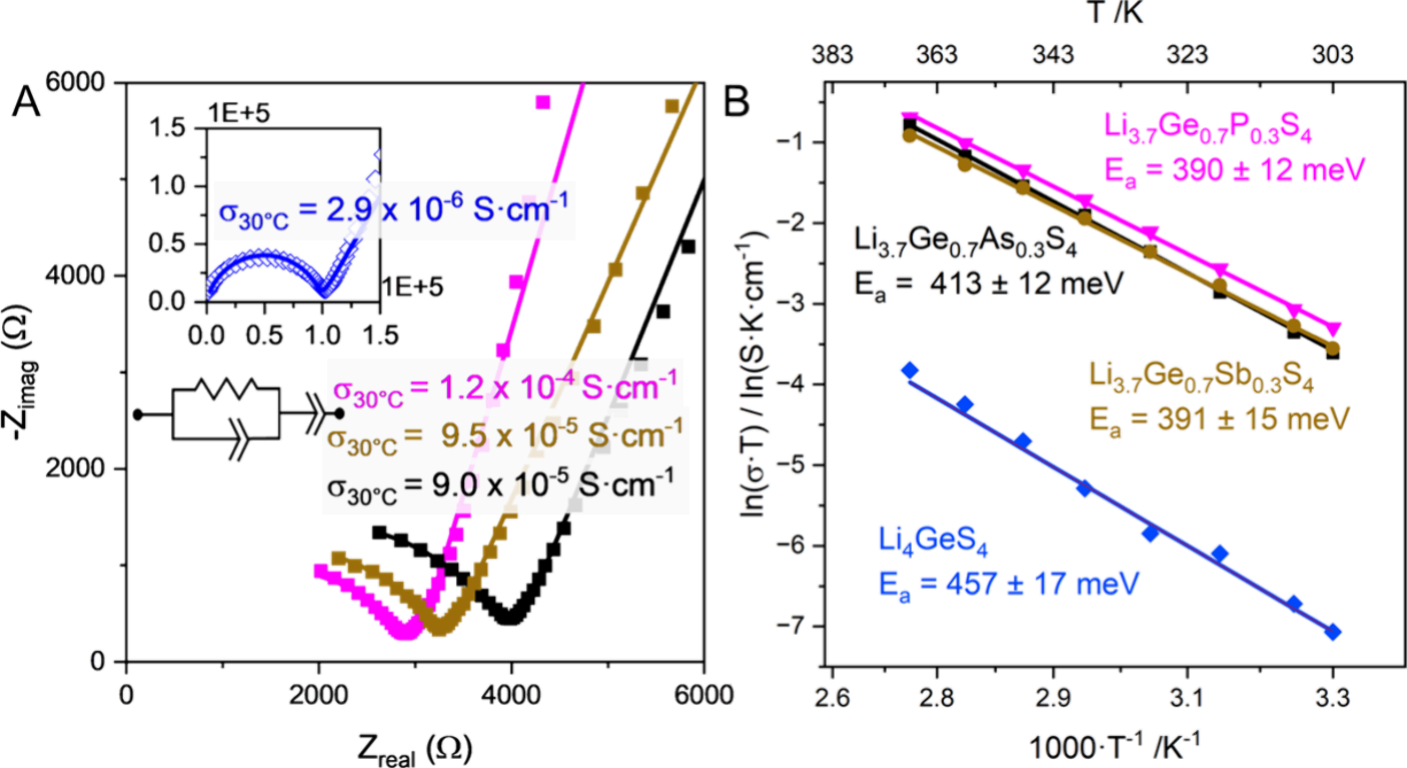
(A) Nyquist plots of the impedance spectra for all materials at 30 °C. The data are shown with markers while the fit is depicted by the solid line. (B) Temperature-dependent ionic conductivity is shown via the Arrhenius plot. The activation energy of Li_4_GeS_4_ is greatly improved with aliovalent substitution of Ge with a pnictogen.

Potentiostatic electrochemical impedance spectroscopy (PEIS) was used to determine the Li^+^ ionic conductivity of Li_4_GeS_4_ and substituted materials. Nyquists plots (Figure 3A) of the impedance spectra exhibit a partial semicircle in the high frequency regime and a relatively linear feature in the low frequency regime. We model these features with a ZARC element (resistor in parallel with a constant phase element (CPE)) in series with a CPE as depicted in the inset of Figure 3A. The ZARC element describes the semicircular feature which can be attributed to ion transport. Within the ZARC element, the value of the resistor is extracted from the fit of the data and used to calculate ionic conductivity. The CPE has a magnitude parameter (Q) and ideality parameter (n) which can be used to calculate an equivalent capacitance, thus informing the type of transport (bulk, grain, interface, etc.) occurring.^22,23^ The ideality parameter of n = 1 would be equivalent to a true capacitor while *n* < 1 indicates a deviation from true capacitive behavior. It is important to note that an ideality parameter that deviates significantly from unity could imply that the model is too simplistic and that more circuit elements need to be added to the equivalent circuit.

Li_4_GeS_4_ was found to exhibit an ionic conductivity (σ_i_) of 2.9 × 10^–6^ S·cm^–1^ at 30 °C under 40 MPa of pressure. The extracted capacitance (11.5 pF) indicates that the (RQ) element of the equivalent circuit is due predominantly to bulk lithium ion conductivity,^23^ but the ideality value of the CPE (0.87) may suggest that another process such as grain boundary conduction may be unresolved. This value of ionic conductivity is within the regime of previous reports of bulk Li_4_GeS_4_ which range from 2.0 × 10^–7^ S·cm^–1^ to 4.0 × 10^–6^ S·cm^–1^.^14,24,25^ It is interesting to note that thin films of Li_4_GeS_4_ have been reported to exhibit conductivities as high as 7.5 × 10^–4^ S·cm^–1^.^26^ This discrepancy may be attributed to different synthetic methods, lack of grain boundaries in the thin films, and a different applied pressure used during measurement of the impedance.^27^

Aliovalent pnictogen substitution in Li_3.7_Ge_0.7_Pn_0.3_S_4_ increases the conductivity by 2 orders of magnitude compared to Li_4_GeS_4_ (Figure 3). The ionic conductivity at 30 °C of all substituted samples is within the range of 9.0 × 10^–5^ to 1.2 × 10^–4^ S·cm^–1^. We extract similar capacitance values (11.5–13.7 pF) and ideality values (0.78–0.79) to those for unsubstituted Li_4_GeS_4_. We therefore assign the value of the resistance from the ZARC circuit element to bulk Li^+^ transport in the substituted analogs.

In order to understand the order-of-magnitude differences in ionic conductivity between Li_4_GeS_4_ and the substituted analogs, we performed temperature-dependent electrochemical impedance spectroscopy. All samples exhibit Arrhenius behavior between *T* = 30 °C–95 °C, as shown in Figure 3B. Li_4_GeS_4_ was found to have an activation energy of 457 ± 17 meV. Pnictogen aliovalent substitution significantly reduces the activation energy for Li^+^ transport. The substituted materials exhibit lower activation energies of 390 ± 12, 413 ± 12, 391 ± 15 meV for P, As, and Sb, respectively. Larger unit cells typically correlate with lower activation energies as there is a widening of the diffusion pathways.^18^ However, Li_3.7_Ge_0.7_P_0.3_S_4_ has nearly the same lattice volume compared to that of Li_4_GeS_4_ yet exhibits enhanced ion conduction and dramatic lowering of the activation energy. Clearly, this does not originate from widening of diffusion pathways. For the substituted Li_4_GeS_4_ system, the Li concentration and Li distribution plays a much larger role in the enhancement of ion conduction. This is further supported by the small changes in conductivity and activation energy between the substituted Li_3.7_Ge_0.7_Pn_0.3_S_4_ compounds.

### 3.4 Bond Valence Site Energy Analysis of Lithium Diffusion Pathways

**Figure 4:**
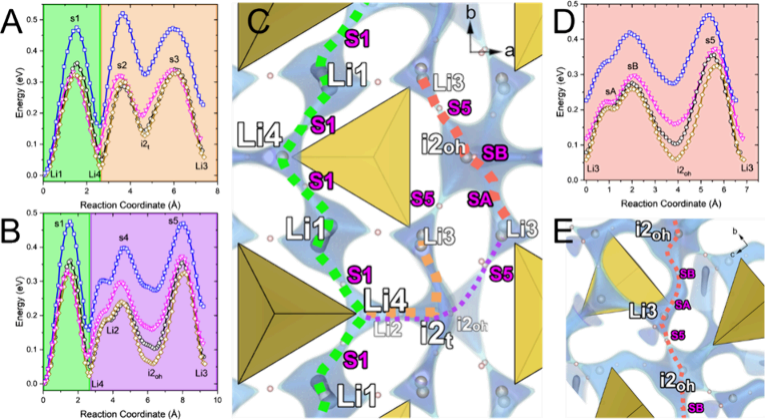
(A, B) Reaction coordinate diagrams showing the ‘Li1–Li4–Li1’ path (green) as well as other connecting paths Li4–i2_t_–Li3 (orange) and Li4–i2_oh_–Li3 (purple) (C) Visualization of Li pathways through Li_3.7_Ge_0.7_P_0.3_S_4_ showing percolation of the Li1–Li4–Li1 pathway. Dotted lines show pathways described in reaction coordinate diagrams. (D, E) Reaction coordinate diagram and visualization for secondary percolation path Li3–i2_oh_–Li3

In order to rationalize the differences in Li^+^ conduction between Li_4_GeS_4_ and the substituted compounds, we analyzed the impact of substitution on the ion diffusion pathways using bond valence site energy (BVSE) analysis implemented in softBV.^28^ The refined structures from our Rietveld refinements were used as the structure inputs and a constant screening factor of 0.75 was applied for all calculations to generate comparable results. The initial distribution of Li occupancies is not important for softBV as it neglects Li^+^-Li^+^ repulsions.^28^ For simplicity, we arbitrarily reduced the Li occupancies equally to generate a charge balanced composition for the Li_3.7_Ge_0.7_Pn_0.3_S_4_ compounds. SoftBV identified 3 interstitial sites relevant to Li conduction pathways which we have named Li4, i2_t_, and i2_oh_. The tetrahedral Li4 site has been identified in previous work as having some fractional Li occupation.^14^ i2t and i2oh are so-named because of their tetrahedral and octahedral coordination, respectively. Two low-energy, percolating, one-dimensional conduction pathways have been identified in Li_4_GeS_4_ and the substituted compounds. The first is the pathway in the *ab* plane involving Li1 and Li4 (Figure 4, green). Given that it is probable that Li4 and Li2 are partially occupied, yet never both occupied at the same time due to their proximity (<1.3 Å), a lower Li2 occupation would seemingly enable lower migration energies due to reduced Li^+^-Li^+^ repulsions. In line with this argument, the energy barrier is dramatically reduced by 115–155 meV from unsubstituted to substituted compositions. This qualitatively agrees with the reduction in activation energies determined from our impedance analysis. Relative differences of ±40 meV between the migration barrier energies for the Li1–Li4 hop is observed between the substituted compounds which qualitatively agrees with the small difference in activation energies determined from PEIS of the substituted compounds. The second one-dimensional percolating conduction pathway is in the *bc* plane involving the Li3 and i2_oh_ site (Figure 4D, E). Here we expect the tetrahedral Li3 site to be mostly occupied and the i2_oh_ site to be mostly vacant. Between these two oct-tet paths, the high energy saddle points (s1, s5) are both located on the center of the shared face between these polyhedra. We found that the distance from the saddle point to the nearest three sulfur atoms did not necessarily correspond with the energy barrier predicted by softBV. For instance, the average s5–sulfur distance in Li_4_GeS_4_ is 2.34 Å while only 2.30 Å in Li_3.7_Ge_0.7_Sb_0.3_S_4_ despite a predicted difference in migration energy of 145 meV. This indicates that the geometrical restraints commonly referred to as the “bottleneck” in this system are not the limiting factor for conduction. Both 1D pathways, ‘Li1–Li4–Li1’ and ‘Li3-i2_oh_-Li3’, have similar energies and are nearly perpendicular to each other, but do not intersect, thus the system may be constrained to diffusion in two dimensions. However, two nonpercolating paths between Li4 and Li3 are possible and have relatively similar energy barriers to the percolating pathways (Figure 4, orange, purple). The first pathway passes through the tetrahedral interstitial site, i2_t_, which connects Li4 to Li3. Alternatively, Li4 is connected by Li2 and the interstitial i2_oh_. Since these nonpercolating pathways connect the two percolating paths in the *ab* and *bc* planes, this material has the potential to exhibit 3D ion conduction, a highly desirable feature for ion conductors.

With these observations it appears that the increase in the Li^+^ vacancy concentration drives the reduced barriers for ion conduction in this system. Aliovalent substitution impacts the anion lattice through subtle shifting of the sulfur positions but does not appear to be the driving factor for the lower migration energies. Expansion of the lattice cannot be the driving factor for the dramatic reduction of activation energy as Li_4_GeS_4_ and Li_3.7_Ge_0.7_P_0.3_S_4_ have nearly the same lattice volume. Additionally, the choice of pnictogen has marginal effects on activation barriers between compounds.

### 3.5 Arrhenius Prefactor and Meyer–Neldel rule

To understand the differences in the Arrhenius prefactor (Table S7) within the Li_3.7_Ge_0.7_Pn_0.3_S_4_ series, it is important to look at the terms complicit in the prefactor (eq 3).^29,30^
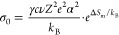
3

The first term of the prefactor contains modifiable parameters: mobile carrier concentration (*c*), jump frequency (ν), jump distance (α), and dimensionality of transport (γ). These terms result in linear changes to the prefactor as opposed to the second term which is exponentially related to the entropy of migration (*S*_m_). When the prefactor changes several orders of magnitude, like in the case of many oxide LISICONs, it is typically attributed to changes in the entropy of migration term.^31−33^ The change in prefactor from Li_4_GeS_4_ to the Li_3.7_Ge_0.7_Pn_0.3_S_4_ series is less than 1 order of magnitude so we cannot assume the change in the entropy of migration to be solely responsible for the majority of the prefactor change. Based on our structure and BVSE analysis, we do not expect changes to the jump distance (α) or dimensionality of transport (γ) from one composition to another. Carrier concentration surely changes from Li_4_GeS_4_ to the Li_3.7_Ge_0.7_Pn_0.3_S_4_ series as vacancies are introduced and the Li lattice purportedly changes, but we cannot definitively determine how many additional mobile charge carriers are activated and if this is enough to justify the total change in prefactor. Additionally, the jump frequency (ν) decreases with increasing lattice softness which also correlates to lowers activation energy.^34^ In agreement with the measured and calculated activation energies, Li_3.7_Ge_0.7_As_0.3_S_4_ has the highest activation energy and prefactor. It is possible this is due to Ge and As being the most similar in terms of ionic radii (0.39 vs 0.34 Å) and causing the least disruption to the anion lattice (seen by most similar average S–S distances SI Table 2.9) and thus the jump frequency is slightly higher than the P and Sb compounds. Ultimately, we identify jump frequency, carrier concentration and entropy of migration as likely candidates for influence on the increasing prefactor of Li_3.7_Ge_0.7_Pn_0.3_S from Li_4_GeS_4_ but further work needs to be done to confidently assert which variables are changing to cause the increase in prefactor.

The substituted Li_3.7_Ge_0.7_Pn_0.3_S_4_ family exhibits both a lower activation energy and a higher Arrhenius prefactor. The activation energy in the substituted materials decreases from unsubstituted Li_4_GeS_4_ by 44–67 meV while the Arrhenius prefactor increases from 3.4 × 10^4^ S·K·cm^–1^ in Li_4_GeS_4_ to 1.1 × 10^5^–2.1 × 10^5^ S·K·cm^–1^ in the Li_3.7_Ge_0.7_Pn_0.3_S_4_ series. The inverse trend between activation barrier and Arrhenius prefactor with substitution appears to violate the Meyer–Neldel rule, which states that a positive correlation between activation energy and Arrhenius prefactor exists and can be supported by the isokinetic theory.^35,36^ However, it is beneficial to violate the Meyer–Neldel rule because high ionic conductivity would benefit from both a low activation energy and a high Arrhenius prefactor. Therefore, violations of Meyer–Neldel should be carefully considered and understood to further inform methods of increasing ionic conductivity. The oxide analogs of the materials in this study exhibit similar deviations from Meyer–Neldel.^31^ While isovalent substitution series between Li_3_PO_4_ and Li_3_VO_4_ follows the expected Meyer–Neldel relationship, once aliovalent substitution with Li_4_GeO_4_ is added, the relationship between decreasing activation energy and decreasing prefactor is seemingly broken. Similar to the relationship between Li_3.7_Ge_0.7_Pn_0.3_S_4_ and Li_4_GeS_4_, Li_3.8_Ge_0.8_P_0.2_O_4_ exhibits a lower activation energy and a higher prefactor than Li_4_GeO_4_. As discussed extensively by Muy et al.,^31^ the Meyer–Neldel (*E*_a_ vs σ_0_) relationship holds within structural families of ion-conductors, but different families of ion-conductors exhibit different slopes and different intercepts. Multiexcitation entropy theory rationalizes the difference in slopes by acknowledging a difference in energy scales which, in the case of ion conduction, can be related to fundamental properties of the crystal lattice such as phonon band center and Debye temperature.^31,37−39^ This connection between the slope in a Meyer–Neldel plot and phonon band center implies that there is a fundamental difference between the structures of Li_4_GeS_4_ and that of Li_3.7_Ge_0.7_Pn_0.3_S_4_. We confirmed through our structural analysis that the anion sublattice of Li_3.7_Ge_0.7_Pn_0.3_S_4_ remains remarkably unchanged to Li_4_GeS_4_ and that the pnictogen substitutes at the Ge site as expected. Therefore, in agreement with the structural analysis the apparent nonconformity to Meyer–Neldel with respect to Li_4_GeS_4_ suggests that a fundamental change to the Li lattice is expected.

### 3.6 Electrochemical Cycling and Critical Current Studies

While many sulfide solid electrolytes exhibit relatively high ionic conductivity, electrochemical stability against anode or cathode materials is problematic in these materials.^40^ The oxidative electrochemical stability of sulfide materials are limited by sulfur, and a thorough investigation of the effects on substitution of the P site in the argyrodite family (Li_6+x_M_*x*_P_1–*x*_S_5_Cl, M = Si, Ge) shows negligible impact on oxidative stability.^41^ We expect that the oxidative stability of thio-LISICONs in this study are similarly constrained. However, the substitution of the main group metal site should affect the reductive stability of the material. While absolute thermodynamic and electrochemical stability plays an important role in the degradation of solid electrolytes, the slow kinetics of the solid-state and interphase formations can also play an important role in the apparent electrochemical stability.^42,43^

We performed critical current density (CCD) tests of the substituted compounds to test their ability to reversibly plate and strip Li and their resistance to forming dendrites.^44^ We constructed symmetric cells of Li|SSE|Li and applied a pressure of 25 MPa which was held for 30 min and subsequently reduced to 5 MPa and maintained throughout the CCD test. This procedure adopted from Ham et al. allowed us to create a conformal Li-SSE interface and yet measure the CCD under a relatively low stack pressure to avoid overinflating the CCD value.^45^ The CCD was then investigated by repeating 10 min chronopotentiometry (CP) experiments with alternating current directions. In between every half cycle a PEIS measurement (∼6 min) was performed to interrogate the evolution of electrochemical processes of the symmetric cell. Additionally, the PEIS verifies that the cell has not shorted.^44^ The current density started at 10 μA·cm^–2^ and was increased by 10 μA·cm^–2^ after every other CP hold until failure or until the cell was too resistive to measure.

**Figure 5:**
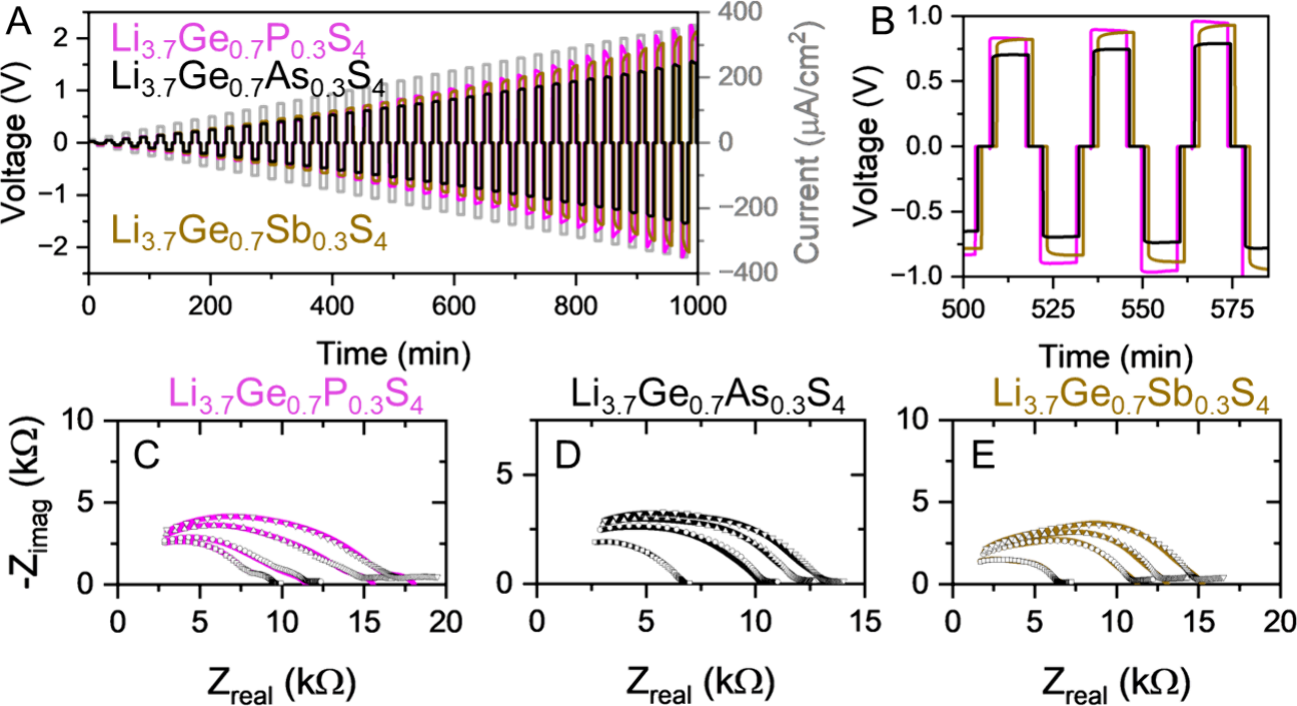
(A) Voltage/current vs time curves for the critical current density tests of Li|SSE|Li symmetric cells for the first 1000 min. (B) Enlarged portion of voltage vs time showing relatively flat voltage features indicating an ionically conductive interface free of Li voiding. (C, D, E) Impedance spectra shown on Nyquist plots for cycles 1, 10, 20, and 30 for P (pink), As (black), and Sb (yellow) substituted materials, respectively. The Nyquists for each material show an evolving interfacial region consistent with growth of a solid electrolyte interphase due to electrochemical instability with Li metal.

Our substituted materials Li_3.7_Ge_0.7_Pn_0.3_S_4_ (Pn = P, As, Sb) all resulted in high critical current densities on par with the argyrodite family, despite an evolving interphase. The unsubstituted Li_4_GeS_4_ was not tested due to its substantially lower ionic conductivity. Such low ionic conductivity would result in large voltages that would not lead to a meaningful comparison. Voltage vs time curves for the CP experiment (Figure 5A) show the expected behavior of increasing voltages and relatively flat voltage profiles before 300 min indicating a conformal interface between Li and the SSE with limited void formation and dendrite growth. All 3 materials lasted over 70 cycles reaching a current density of *j* > 0.7 mA·cm^–2^ and all samples became too resistive to measure rather than shorting. These CCD values are on par with the argyrodite, Li_6_PS_5_Cl, which has a wide variance in reported values from 0.1–2.15 mA·cm^–2^ under a range of pressure/cell conditions.^45,46^ However, a practical CCD of Li_3.7_Ge_0.7_Pn_0.3_S_4_ materials would be much lower than 0.7 mA·cm^–2^, as the voltage generated is large and serious degradation of the material is expected during prolonged cycling. However, this could be mitigated by adjusting thickness of the electrolyte, which in turn could impact the resistance to dendrites. Despite this interplay, our results highlight the primary issue of Li_3.7_Ge_0.7_Pn_0.3_S_4_ to be related to the interphase formation.

Pnictogen substitution changes the decomposition products that determine whether the interphase region will be stable (ion conduction only) or whether it will propagate (mixed ion-electron conduction) through the SSE. Analysis of the PEIS unveiled that initially all samples formed good conformal contact with Li metal (Figure 5C-E). The first impedance spectrum on all samples shows at least two partially convoluted semicircle features. This is consistent with the high frequency (low Z_real_) feature being responsible for bulk ion conduction and the second feature corresponding to interfacial impedance of the solid electrolyte with a Li electrode. We attempted to fit all spectra with two ZARC elements in series but found that the obtained fits were especially poor beyond 20 cycles. Subsequently, we found that three ZARC elements in series yielded a much better fit of the data indicating that at least three major ion transport processes may be occurring. However, we note that these processes overlap significantly in the Nyquist plots, which makes it challenging to model. We therefore adopted a constrained refinement approach to establishing realistic boundary conditions for each ZARC element. We assigned the first ZARC element to bulk ionic conductivity and constrained the resistance close to the expected values for ionic conductivity which agrees with the main feature we see in the initial impedance spectrum. The capacitance values obtained from this first ZARC element are similar to those observed from PEIS with blocking electrodes (∼10^–11^ F) and are consistent with bulk ion conduction. We assign the second and third ZARC elements to the interfacial charge transport processes between SSE/SEI and the SEI/Li, respectively. Because the two interfacial processes occur on similar time scales it is difficult to deconvolute these features and impose restrictions on capacitance and resistance of these features. Collectively they are also changing dramatically over the course of the experiment (Figure S14). From the first cycle to the last cycle, PEIS shows a continual increase in the real and imaginary impedance of the low frequency semicircle for all samples. This growth of the low frequency features is consistent with an interface that is evolving over time through the formation of a SEI. Our CCD test clearly exhibits a growing voltage profile and growing interfacial impedance for all samples, suggesting that no stable interface is ever achieved in any of the substituted materials. In conclusion, the substitution of P, As, and Sb do not contribute to formation of a stable SEI. Because all substituted samples have similar ionic conductivity, the overall impedance and maximum current densities are also similar. While the SEI growth is problematic for stable long-term cycling with Li metal, we find that aliovalent substitutions are necessary to increase ionic conductivity and reduce the cell overpotential during cycling.

### 3.7 Probing the Interphase through Virtual Electrode–X-ray Photoelectron Spectroscopy

To further probe the chemical reactivity and evolution of the interphase during cycling against Li metal we performed a virtual electrode X-ray photoelectron spectroscopy (VE-XPS) experiment on all substituted materials. In VE-XPS, the solid electrolyte is placed in contact with Li metal foil and electrically grounded to the instrument. Bombarding the surface with low-energy electrons (10 eV kinetic energy, current density ∼10 μA/cm^2^) provides a negative surface charge density that drives Li^+^ migration to the surface. Once Li^+^ reaches the surface it undergoes reduction to form SEI phases and then, in cases where the SEI passivates with respect to electron conduction, eventually Li^0^.^47,48^ Between pulses of electron flux, the surface is analyzed through XPS and provides information about the evolution of the interface between the plated Li and the electrolyte.

**Figure 6:**
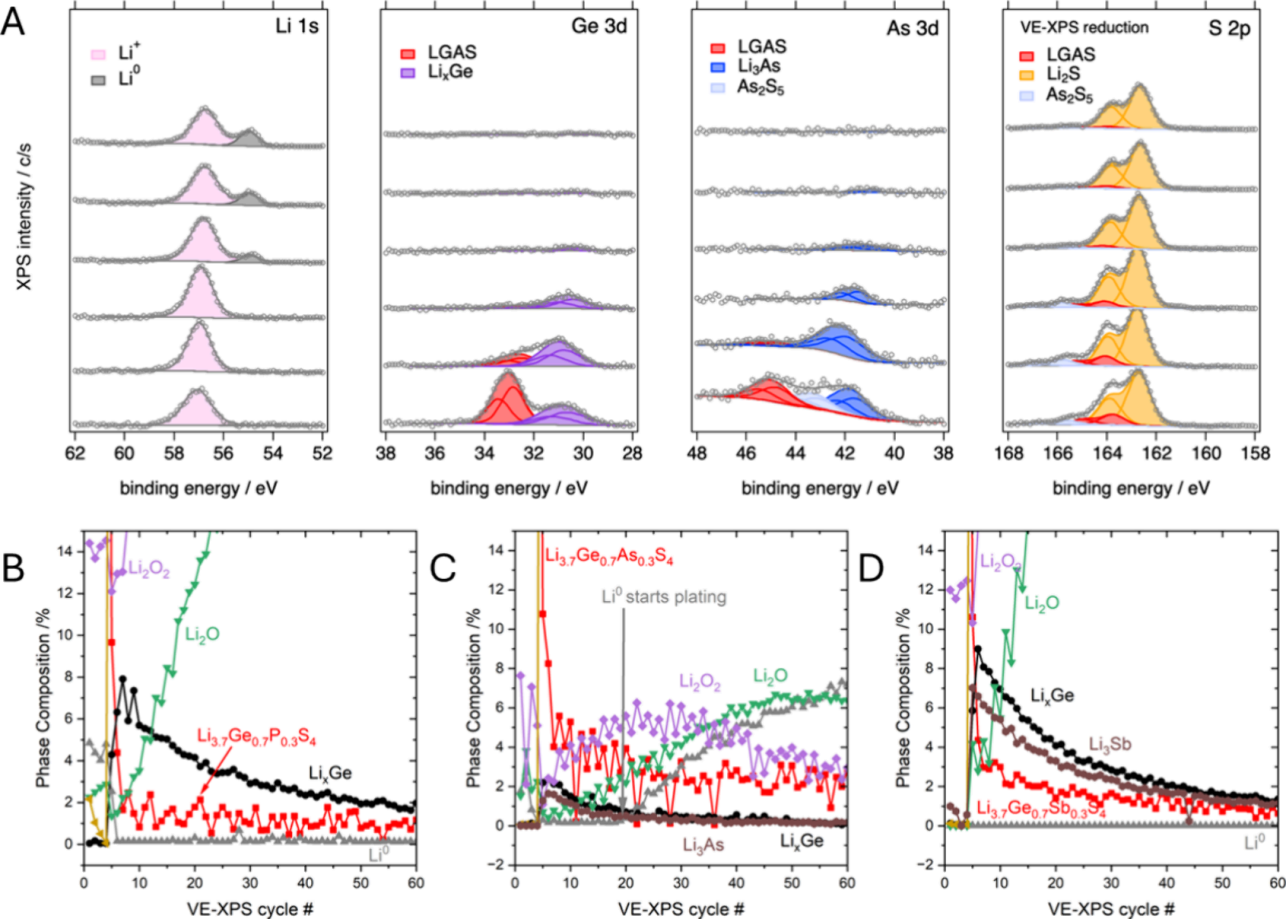
(A) Selected XPS spectra from virtual electrode experiment on Li_3.7_Ge_0.7_As_0.3_S_4_. Li plating can be seen after ∼20 cycles and the surface of the electrolyte is dominated by Li_2_S. (B–D) Zoomed in phase composition (%) as a function of VE-XPS cycle for P, As, Sb substituted materials, respectively. For clarity, contributions from adsorbed C species (<5%) have been omitted.

VE-XPS reveals an evolving interphase in agreement with the growing impedances and cell voltages observed from the CCD tests. Unlike CCD, XPS allows the identification of the decomposition products of Li_3.7_Ge_0.7_Pn_0.3_S_4_. Upon reduction against Li^+^/Li^0^ we observe formation of Li_2_S, Li_*x*_Ge, and Li_3-x_Pn (Figure 6) with two exceptions. First, we never observe a Li_3-x_Pn phase for the compound substituted with P. We justify this by pointing to a similar experiment performed on Li_10_GeP_2_S_12_, which also sees a quick disappearance of reduced P species, even when performed at −80 °C where the kinetics should be much slower and the content of P is much higher than in our sample.^49^ Second, despite our best efforts to minimize water and oxygen in the sample, we observe the formation of Li_2_O and Li_2_O_2_ presumably from small concentrations of H_2_O and O_2_ in the sample chamber or contaminants from the brief exposure to atmosphere when loading the sample in the XPS.^48^ In the samples containing P and Sb the large amounts of LiOH and Li_2_O_2_ hinder our ability to observe plating of Li^0^.

Upon starting the VE-XPS experiment, the SEI is immediately formed and dominated by Li_2_S in all samples. Accompanying this change is the formation of Li_3_Pn and Li_*x*_Ge as well as a dramatic decrease of the starting material in the surface region being probed. In all cases, Li_2_O and Li_2_O_2_ phases start to grow after about 5 “cycles”; here, we define an XPS “cycle” as an electron pulse followed by a XPS measurement. In the P and Sb case, the oxide compounds become the dominant feature of the interphase which we would not expect to be representative of what occurs during our cycling experiments. In contrast, Li_3.7_Ge_0.7_As_0.3_S_4_ starts to plate Li metal after ∼ 20 cycles. Figure 6A shows the Li metal (binding energy ∼ 55 eV) peak for Li_3.7_Ge_0.7_As_0.3_S_4_ growing as a function of cycle number. The plating of Li metal suggests that the surface of the electrolyte is passivated. Indeed the SEI is dominated by the growth of Li_2_S which is known to be an electronic insulator with a low ionic conductivity ∼ 10^–9^ S cm^–1^ and therefore could form a passivating interphase.^50,51^ However, given that metallic Li plating at the surface is not observed until ∼ 20 XPS cycles for Li_3.7_Ge_0.7_As_0.3_S_4,_ and never for Li_3.7_Ge_0.7_P_0.3_S_4_ and Li_3.7_Ge_0.7_Sb_0.3_S_4_, coupled with monotonic decreases of Li_3.7_Ge_0.7_Pn_0.3_S_4_ phase fractions and increases in resistance during cycling, we suggest that continual SEI propagation occurs in all samples. To fully understand this, we must consider the other phases in the complex SEI.

According to the proposed balanced chemical equation (eq 4) of the decomposition products, Li_2_S should be responsible for 80 mol % of our interphase region. Calculating the phase
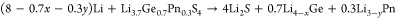
4

composition from the XPS spectra in the case of Li_3.7_Ge_0.7_As_0.3_S_4_ yielded a near exact match of this value (Figure S19). For the P and Sb versions, the results are impacted by the oxide phases but are consistent with Li_2_S being the dominant decomposition product. The decomposition products involving P and Ge should be the same as those reported for Li_10_GeP_2_S_12_. A computational investigation of the electrochemical stability of Li_10_GeP_2_S_12_ shows that P^5+^ is the first species to be reduced, followed by Ge^4+^. Both P and Ge are first reduced to their elemental forms before additional reduction leads to lithiated phases.^52^ Ge^0^ and reduced Li_*x*_Ge are electronic conductors and should be the second most prominent phase in the interphase when considering the balanced chemical equation of decomposition. Ge phases have been attributed in Li_10_GeP_2_S_12_ to the rapid growth of the SEI when compared to a Ge-free phase, Li_7_P_3_S_11_.^49,53^ Interestingly, after initial SEI formation, Li_10_GeP_2_S_12_ also exhibits a similar slowly propagating SEI that is ultimately attributable to the presence of electronically conductive Ge phases.^49^ Here, we suggest that the continual SEI formation observed in this work is consistent with that observed in Li_10_GeP_2_S_12_ and that the underlying mechanism of the propagation of SEI is driven by electronic leakage. Changing the pnictogen in this system does not substantially impact the propagation of the SEI primarily because it is the smallest component of a complex interphase region and the presence of a conducting phase in all samples excludes the possibility of a nonpropagating interphase. Ultimately, the VE-XPS experiments correlate with our cycling experiments to show that Ge and its derivative phases are responsible for the propagation of the SEI.

## 4 Conclusion

In this work we explore the aliovalent substitution series of Li_4_GeS_4_, Li_3.7_Ge_0.7_Pn_0.3_S_4_ (Pn = P, As, Sb), and characterize the relative changes in the structure, ionic conductivity, activation energy, and stability against Li metal. HR-PXRD confirms incorporation of the pnictogens and the nominal composition of Li_3.7_Ge_0.7_Pn_0.3_S_4_. Furthermore, each substituted material shows an anisometric expansion of the unit cell with a shortening of the *a* lattice parameter and lengthening of *b*. Although the unit cell volumes are different due to the substituted pnictogen, all substituted compounds exhibit nearly the same *a*/*b* ratio. X-ray PDF shows that there is no additional local ordering of the Ge-Pn cations, suggesting that the anisometric expansion of the unit cell is due to redistribution of the Li atoms in the unit cell.^14^ As the size of the substituted pnictogen increases from P → As → Sb, the volume of the unit cell increases accordingly, leading to a slight lowering of the activation energy for Li_3.7_Ge_0.7_Sb_0.3_S_4_ as determined by temperature-dependent PEIS. However, unit cell volume does not explain the dramatic reduction in activation energy for the substituted analogs versus Li_4_GeS_4_. BVSE calculations corroborate the observed similarity of activation energies in Li_3.7_Ge_0.7_Pn_0.3_S_4_ samples and insinuate that changes in the Li sublattice drive changes in the activation energy. Analysis of the Arrhenius prefactor also suggests a rearrangement of the Li lattice to be complicit in the seeming disruption of the Meyer–Neldel rule. This conclusion reinforces the need for high quality structural characterization of solid electrolytes and especially the Li^+^ sublattice, as small changes in the crystal structure can have significant impacts on ion conduction.

Beyond ionic conductivity, the choice of the pnictogen has limited impact on electrochemical stability and solid electrolyte interphase formation with lithium metal. Although Li_3.7_Ge_0.7_As_0.3_S_4_ showed Li plating in the VE-XPS experiment while samples with P and Sb did not, the decrease in phase fraction of Li_3.7_Ge_0.7_Pn_0.3_S_4_ throughout the experiment suggests that all phases studied form unstable interphases that continually grow due to the presence of reduced Ge species. PEIS throughout the CCD experiments also show continual growth of the interphase and increasingly more resistive cells. Despite reductive stability being an issue, Li_3.7_Ge_0.7_Pn_0.3_S_4_ forms good interfacial contact with lithium metal and the high lithium-ion conductivities afforded by aliovalent substitution suppresses dendrite formation even at relatively high current densities.

## Supporting Information Available

XRD, PDF, tables of refined crystallographic parameters and bond distances, temperature dependent Nyquist plots, chronopotentiometry experiments, and virtual electrode XPS results (PDF)
