# Supplementary material for: Directing Ion Transport and Interfacial Chemistry in Pnictogen-Substituted Thio-LISICONs
Source: ACS Appl Mater Interfaces. 2025 Mar 20;17(13):19906–16. doi: 10.1021/acsami.4c22390 (PMC11969426; doi:10.1021/acsami.4c22390)
Supplement: Supplementary file 1 — am4c22390_si_001.pdf [file am4c22390_si_001.pdf]

Supporting Information

Directing Ion Transport and Interfacial Chemistry in Pnictogen-Substituted Thio-LISICONs

Philip Yox[a,b], Glenn Teeter[b], Lucas Baker[a], Drew Whitney[a], Annalise E. Maughan[a,b,*]

[a]Department of Chemistry, Colorado School of Mines, Golden, Colorado 80401, United States

[b]National Renewable Energy Laboratory, Golden, Colorado 80401, United States

[*]Corresponding author: amaughan@mines.edu

Supporting Information Table of Contents

Structure Note *3*

High Resolution XRD Figures and Tables *4-11*

Figure S1 Li_4GeS_4 HR XRD *4*

Figure S2 $Li_{3.7}Ge_{0.7}P_{0.3}S_4$ HR XRD *5*

Figure S3 $Li_{3.7}Ge_{0.7}As_{0.3}S_4$ HR XRD *6*

Figure S4 $Li_{3.7}Ge_{0.7}Sb_{0.3}S_4$ HR XRD *7*

Table S1 Lattice Parameters and Unit Cell Volumes Table *8*

Table S2 Rietveld Refinement Results Table *8*

Table S3 Li Polyhedral Volumes Table *9*

Table S4 Table of Atomic Coordinates from Rietveld Refinement *10*

Table S5 Table of S-S distances from Rietveld and PDF analysis *11*

Pair-Distribution Function Figures *11-14*

Figure S5 Li_4GeS_4 PDF *11*

Figure S6 $Li_{3.7}Ge_{0.7}P_{0.3}S_4$ PDF *12*

Figure S7 $Li_{3.7}Ge_{0.7}As_{0.3}S_4$ PDF *13*

Figure S8 $Li_{3.7}Ge_{0.7}Sb_{0.3}S_4$ PDF *14*

Electrochemical Impedance Spectroscopy Figures and Tables *15-20*

Figure S9 Li_4GeS_4 Temperature dependent EIS *15*

Figure S10 $Li_{3.7}Ge_{0.7}P_{0.3}S_4$ Temperature dependent EIS *16*

Figure S11 $Li_{3.7}Ge_{0.7}As_{0.3}S_4$ Temperature dependent EIS *17*

Figure S12 $Li_{3.7}Ge_{0.7}Sb_{0.3}S_4$ Temperature dependent EIS *18*

Table S6 EIS fitting parameter Table *19*

Table S7 Table of Activation Energies and Arrhenius Prefactor *20*

Critical Current Density Figures *21-23*

Figure S13 Chronopotentiometry Voltage/Current vs. Time *21*

Figure S14 Evolution of PEIS with Li symmetric cycling *22*

Figure S15 Equivalent circuit for PEIS of CCD test *23*

Table S8 Table of extracted values from evolution of PEIS fits *23*

VE-XPS Figures *24-27*

Figure S16 $Li_{3.7}Ge_{0.7}P_{0.3}S_4$ VE-XPS *24*

Figure S17 $Li_{3.7}Ge_{0.7}As_{0.3}S_4$ VE-XPS *25*

Figure S18 $Li_{3.7}Ge_{0.7}Sb_{0.3}S_4$ VE-XPS *26*

Figure S19 Phase Composition vs. XPS cycle number *27*

Structure Notes

Matsushita and Kanatzidis determined the structure of Li_4GeS_4 from a single crystal X-ray diffraction experiment.[1] Matsushita and Kanatzidis defined Li1 as 8*d* octahedral site (split position with occupancy set to 0.5), Li2 as 4*c* tetrahedral site, and Li3 as 8*d* tetrahedral site. Murayama et al. determined the structure from Rietveld refinement of neutron diffraction data.[2] Murayama et al. defined Li1 as the 4*c* tetrahedral site, Li2 as 8*d* tetrahedral site, and Li3 as a 4*b* octahedral site with full occupancy. A large B_{eq} value of 3.5 Å^2 was used for the Li3 site in the refinement. Because this is a rather large value and the offset octahedral Li position used by Matsushita and Kanatzidis is only ~0.24 Å from the Li position used by Murayama et al., the split position of Li and the atom names from the Matsushita and Kanatzidis structure will be used for our structure refinements.

References

(1) Matsushita, Y.; Kanatzidis, M. G. Synthesis and Structure of Li_4GeS_4. *Z. Naturforsch. B* **1998**, *53* (1), 23–30. https://doi.org/10.1515/znb-1998-0107.

(2) Murayama, M.; Kanno, R.; Kawamoto, Y.; Kamiyama, T. Structure of the Thio-LISICON, Li_4GeS_4. *Solid State Ion.* **2002**, *154–155*, 789–794. https://doi.org/10.1016/S0167-2738(02)00492-7.

High Resolution XRD Figures and Tables

Figure S1: Li_4GeS_4

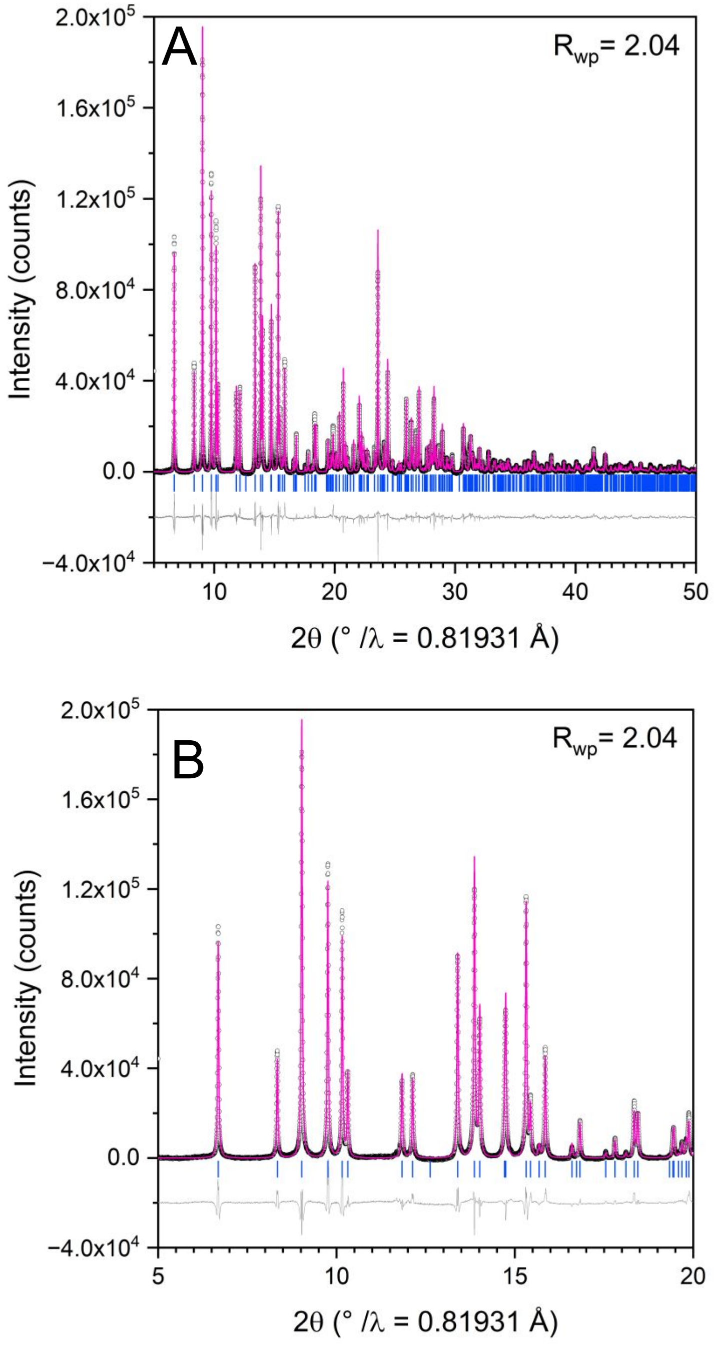

Figure Sl: (A) Rietveld refinement of high-resolution powder diffractogram of Li_4GeS_4. **(B)** Zoomed in region from 2θ = 5-20°. Data, fit, reflections, and difference are shown in black, pink, blue, and gray, respectively.

Figure S2 $Li_{3.7}Ge_{0.7}P_{0.3}S_4$

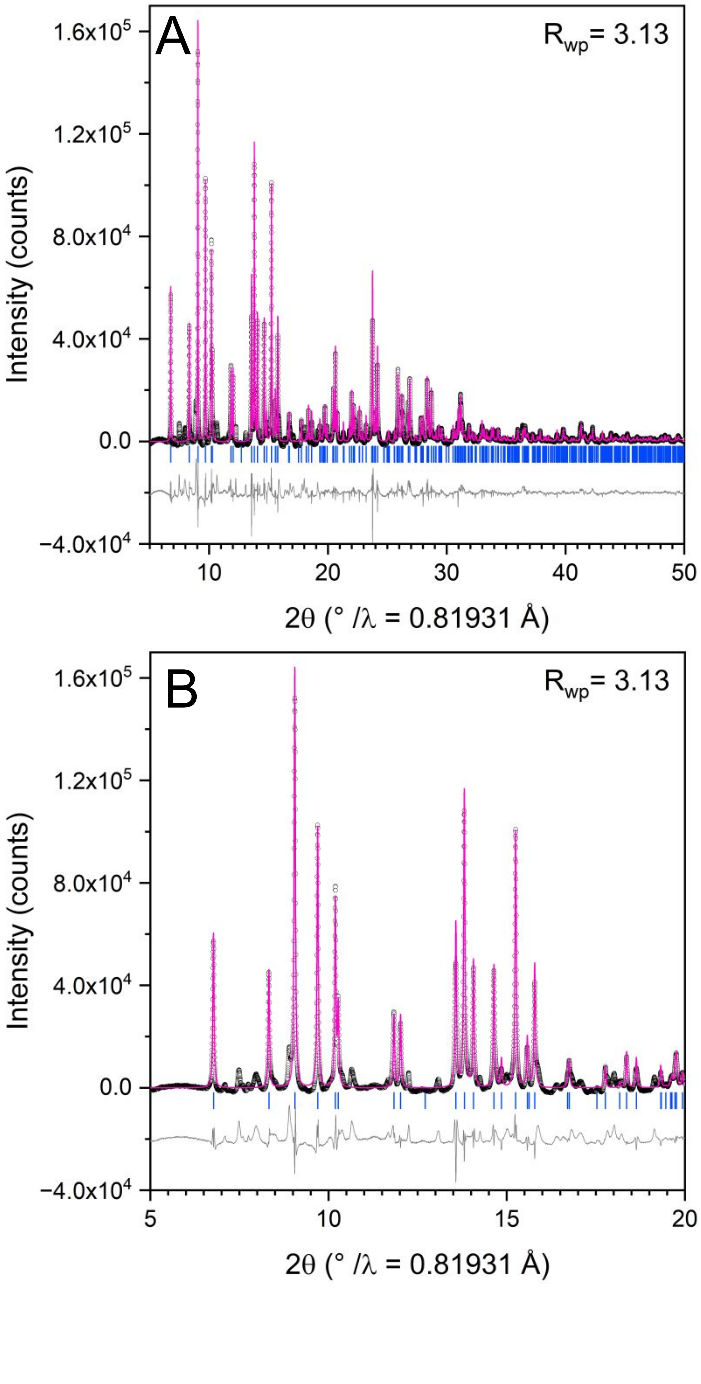

Figure S2: **(A)** Rietveld refinement of high-resolution powder diffractogram of $Li_4Ge_{0.7}P_{0.3}S_4$. **(B)** Zoomed in region from 2θ = 5-20°. Data, fit, reflections, and difference are shown in black, pink, blue, and gray, respectively.

Figure S3 $Li_{3.7}Ge_{0.7}As_{0.3}S_4$

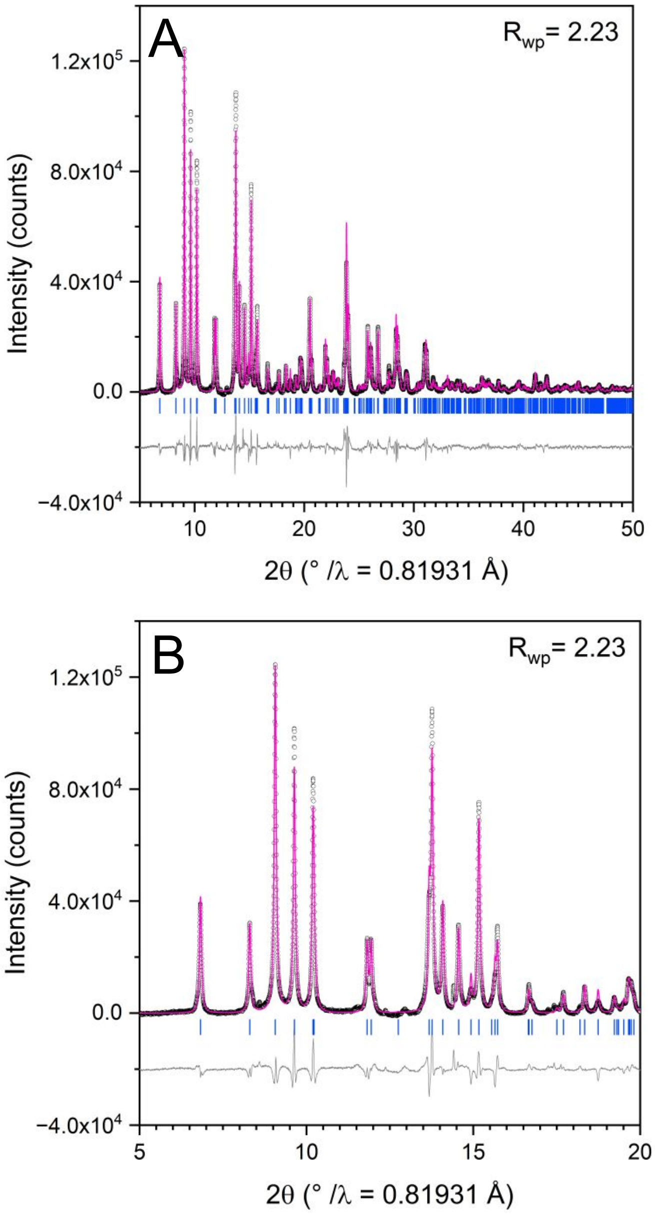

Figure S3: (A) Rietveld refinement of high-resolution powder diffractogram of $Li_4Ge_{0.7}As_{0.3}S_4$. **(B)** Zoomed in region from 2θ = 5-20°. Data, fit, reflections, and difference are shown in black, pink, blue, and gray, respectively.

Figure S4 $Li_{3.7}Ge_{0.7}Sb_{0.3}S_4$

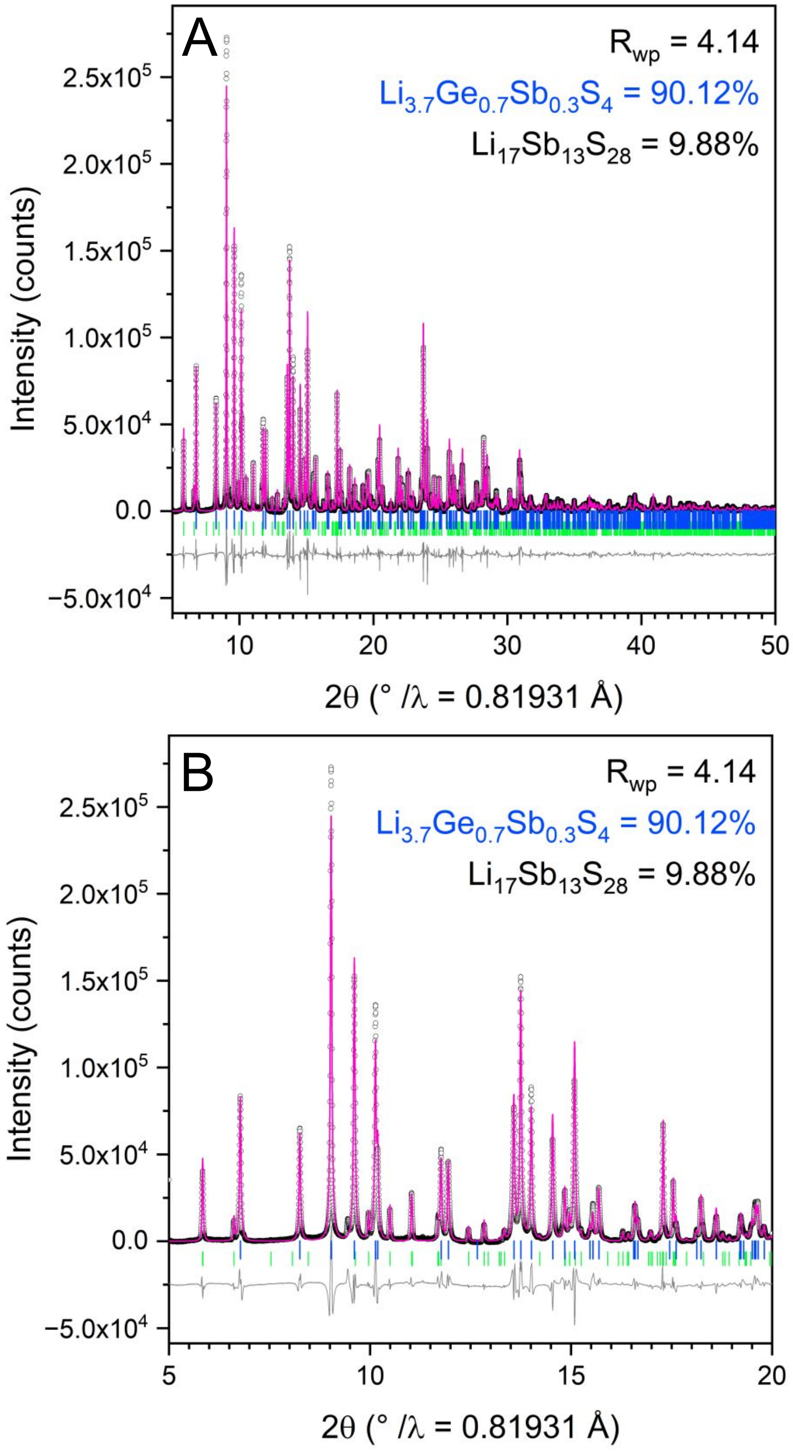

Figure S4: (A) Rietveld refinement of high-resolution powder diffractogram of $Li_4Ge_{0.7}Sb_{0.3}S_4$. **(B)** Zoomed in region from 2θ = 5-20°. Blue and green ticks correspond to $Li_4Ge_{0.7}Sb_{0.3}S_4$ and $Li_{17}Sb_{13}S_{28}$ reflections, respectively. Data, fit, and difference curves are shown in black, pink, blue, and gray, respectively.

Table S1 Lattice Parameters and Unit Cell Volumes Table

	Li_4GeS_4			$Li_{3.7}Ge_{0.7}P_{0.3}S_4$	
	Literature	Rietveld	PDF	Rietveld	PDF (1-10Å)
a (Å)	14.107(6)	14.0477	14.0583	13.8658(1)	13.8638
b (Å)	7.770(3)	7.7501	7.75457	7.82336(6)	7.84113
c (Å)	6.162(2)	6.1473	6.16678	6.17362(4)	6.17788
V (Å^3)	675.43	669.270	672.278	669.701(9)	671.584
a/b	1.81	1.81	1.81	1.77	1.77
		$Li_{3.7}Ge_{0.7}As_{0.3}S_4$		$Li_{3.7}Ge_{0.7}Sb_{0.3}S_4$	
		Rietveld	PDF (1-10Å)	Rietveld	PDF (1-10Å)
a (Å)		13.7596(4)	13.8111	13.8589(1)	13.7844
b (Å)		7.8757(2)	7.85536	7.8690(1)	7.83003
c (Å)		6.2074(1)	6.22145	6.23913(5)	6.24851
V (Å^3)		672.677	674.972	680.414	674.415
a/b		1.75	1.76	1.76	1.76

Table S1: Table of lattice parameters, volumes, and a/b lattice parameter ratio as found in literature and determined from Rietveld and PDF refinements.

Table S2 Rietveld Refinement Results Table

	Li_4GeS_4	$Li_{3.7}Ge_{0.7}P_{0.3}S_4$	$Li_{3.7}Ge_{0.7}As_{0.3}S_4$	$Li_{3.7}Ge_{0.7}Sb_{0.3}S_4$
Radiation (Å)	0.81931			
a (Å)	14.04771(5)	13.8658(1)	13.7596(4)	13.8589(1)
b (Å)	7.75012(3)	7.82336(6)	7.8757(2)	7.8690(1)
c (Å)	6.14734(2)	6.17362(4)	6.2074(1)	6.23913(5)
V (Å^3)	669.27	669.701	672.677	680.414
R_p	1.319	2.253	1.385	2.803
R_{wp}	2.039	3.302	2.226	4.144
R_{bragg}	1.41	1.56	1.23	1.97
# refined parm.	44	51	56	51
# bkg parm.	26	28	36	22
Bkg function	Chebyshev			

Table S2: Table of refined lattice parameters and relevant parameters from Rietveld refinements.

Table S3 Li Polyhedral Volumes Table (from Rietveld)

	Li_4GeS_4	$Li_{3.7}Ge_{0.7}P_{0.3}S_4$	$Li_{3.7}Ge_{0.7}As_{0.3}S_4$	$Li_{3.7}Ge_{0.7}Sb_{0.3}S_4$
Li(1) V (Å^3)	25.5942	27.3938	26.3307	27.1999
Li(2) V (Å^3)	7.2988	7.5547	7.5404	7.7211
Li(3) V (Å^3)	7.5405	7.52765	7.5359	7.4366

Table S3: Table of Li polyhedral volumes as determined from the structures refined by the Rietveld method.

Table S4: Atomic coordinates from Rietveld refinement.

Atom	site mult.	x	y	z	occ	U_{iso} (Å^2)
Li_4GeS_4						
Ge1	4	0.58806	0.75	0.14883	1	2.04
S1	4	0.58543	0.75	-0.2067	1	3.3
S2	8	0.65437	0.98473	0.2719	1	2.02
S3	4	0.43787	0.75	0.2675	1	1.58
Li1	8	0.5022	0.9857	0.5262	0.5	1
Li2	4	0.41167	0.75	-0.1294	1	1
Li3	8	0.67765	0.49997	-0.30753	1	1
$Li_{3.7}Ge_{0.7}P_{0.3}S_4$						
Ge1	4	0.58596	0.75	0.1498	0.7	2.11
P1	4	0.58596	0.75	0.1498	0.3	2.11
S1	4	0.5919	0.75	-0.1953	1	5.77
S2	8	0.65606	0.9757	0.2625	1	4.25
S3	4	0.4420	0.75	0.2655	1	6.07
Li1	8	0.5022	0.9857	0.5262	0.5	1
Li2	4	0.41167	0.75	-0.1294	1	1
Li3	8	0.67765	0.49997	-0.30753	1	1
$Li_{3.7}Ge_{0.7}As_{0.3}S_4$						
Ge1	4	0.58695	0.75	0.15616	0.7	2.41
As1	4	0.58695	0.75	0.15616	0.3	2.41
S1	4	0.5861	0.75	-0.1961	1	3.35
S2	8	0.66219	0.9804	0.2669	1	0.98
S3	4	0.441	0.75	0.2737	1	1.25
Li1	8	0.5022	0.9857	0.5262	0.5	1
Li2	4	0.41167	0.75	-0.1294	1	1
Li3	8	0.67765	0.49997	-0.30753	1	1
$Li_{3.7}Ge_{0.7}Sb_{0.3}S_4$						
Ge1	4	0.58793	0.75	0.15224	0.721	2.50
Sb1	4	0.58793	0.75	0.15224	0.279	2.50
S1	4	0.5901	0.75	-0.2040	1	3.65

S2	8	0.65984	0.9854	0.2685	1	1.13
S3	4	0.4345	0.75	0.2706	1	2.92
Li1	8	0.5022	0.9857	0.5262	0.5	1
Li2	4	0.4515	0.75	-0.098	1	1
Li3	8	0.67765	0.49997	-0.30753	1	1

Table S4: Table of atomic coordinates, occupancy, and isotropic displacement parameter (U_{iso}) determined from Rietveld refinements.

Table S5: S-S distances from Rietveld and PDF analysis

S-S distances	Li_4GeS_4		$Li_{3.7}Ge_{0.7}P_{0.3}S_4$		$Li_{3.7}Ge_{0.7}As_{0.3}S_4$		$Li_{3.7}Ge_{0.7}Sb_{0.3}S_4$	
Distance (Å)	Rietveld	PDF (1-10Å)	Rietveld	PDF (1-10Å)	Rietveld	PDF (1-10Å)	Rietveld	PDF (1-10Å)
S1-S2 (intra)	3.592	3.626	3.449	3.573	3.557	3.560	3.613	3.582
S1-S3 (intra)	3.577	3.636	3.523	3.703	3.534	3.696	3.663	3.516
S2-S2 (intra)	3.638	3.665	3.532	3.720	3.629	3.662	3.705	3.905
S2-S3 (intra)	3.544	3.571	3.454	3.537	3.544	3.550	3.631	3.745
Average	3.588	3.625	3.490	3.633	3.566	3.617	3.653	3.687
S1-S2 (inter)	3.811	3.764	4.074	4.090	3.937	3.878	3.898	3.767
S1-S2 (inter)	3.967	4.031	4.110	3.991	4.069	3.832	4.047	4.192
S1-S3 (inter)	3.840	3.929	3.925	3.969	3.985	4.006	3.971	3.950
S1-S3 (inter)	3.907	3.916	3.964	3.744	3.849	3.986	3.924	3.516
S2-S3 (inter)	3.731	3.707	4.133	3.722	3.828	3.742	3.783	4.090
S2-S2 (inter)	4.089	4.030	4.057	4.044	3.946	3.979	4.004	4.040
Average	3.891	3.896	4.044	3.927	3.936	3.904	3.938	3.926

Table S5: Table of S-S distances determined from Rietveld and PDF refinements. Average intra S-S distances correlates with size of the central atom (Ge/Pn), while inter S-S distances change marginally.

Pair-Distribution Function Figures

Figure S5 Li_4GeS_4

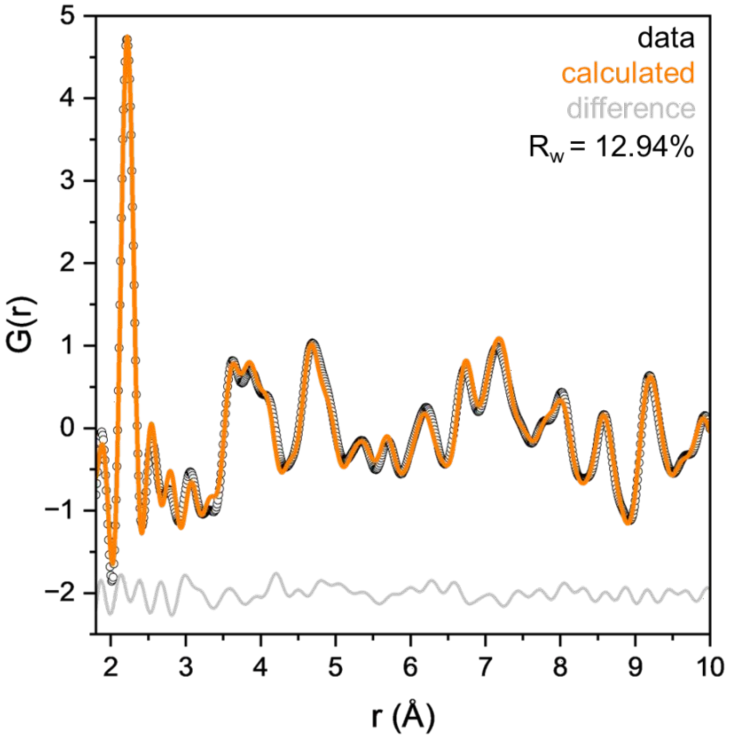

Figure S5: Fitting of the X-ray pair distribution function G(r) of Li_4GeS_4 shows good agreement with the average structure determined from PXRD. Data, calculated model, and difference curve are shown in black, orange and gray, respectively.

Figure S6: $Li_{3.7}Ge_{0.7}P_{0.3}S_4$

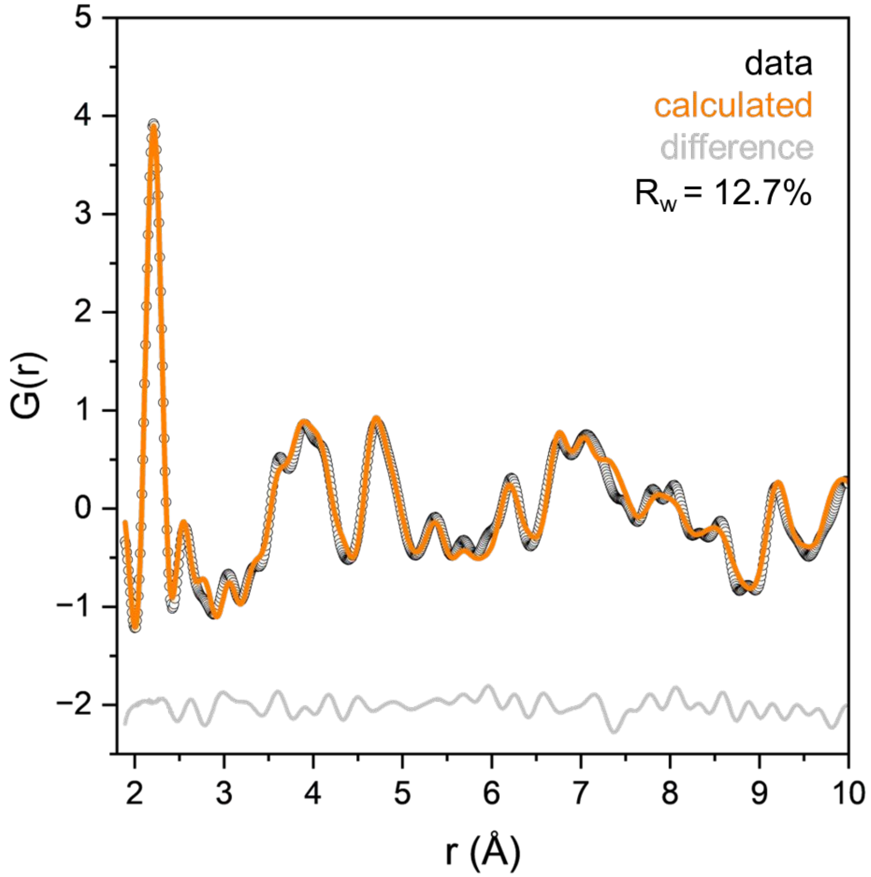

Figure S6: Fitting of the X-ray pair distribution function G(r) of $Li_4Ge_{0.7}P_{0.3}S_4$ shows good agreement with the average structure determined from PXRD. Data, calculated model, and difference curve are shown in black, orange and gray, respectively.

Figure S7: $Li_{3.7}Ge_{0.7}As_{0.3}S_4$

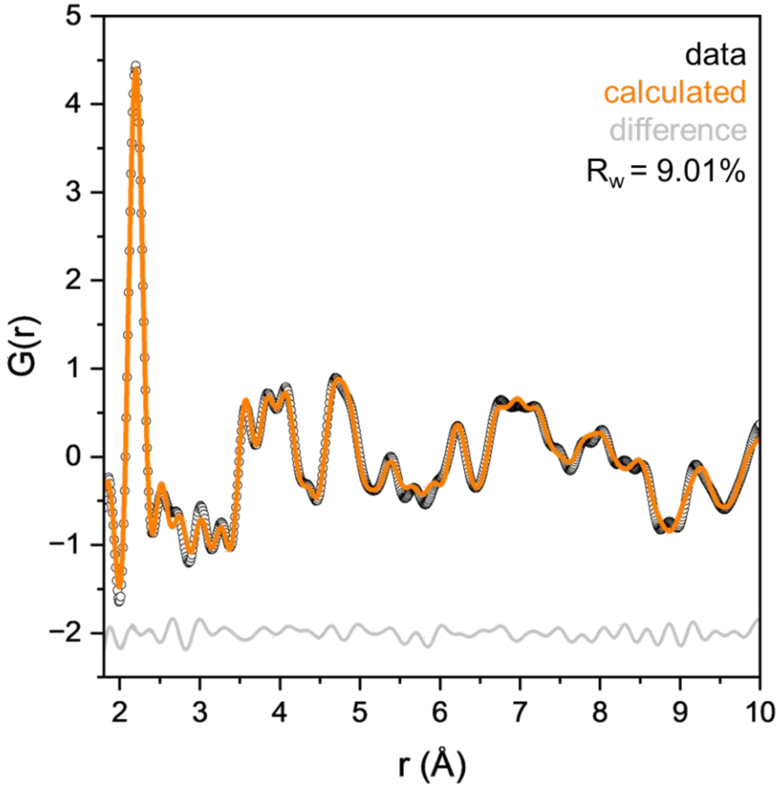

Figure S7: Fitting of the X-ray pair distribution function G(r) of $Li_4Ge_{0.7}As_{0.3}S_4$ shows good agreement with the average structure determined from PXRD. Data, calculated model, and difference curve are shown in black, orange and gray, respectively.

Figure S8: $Li_{3.7}Ge_{0.7}Sb_{0.3}S_4$

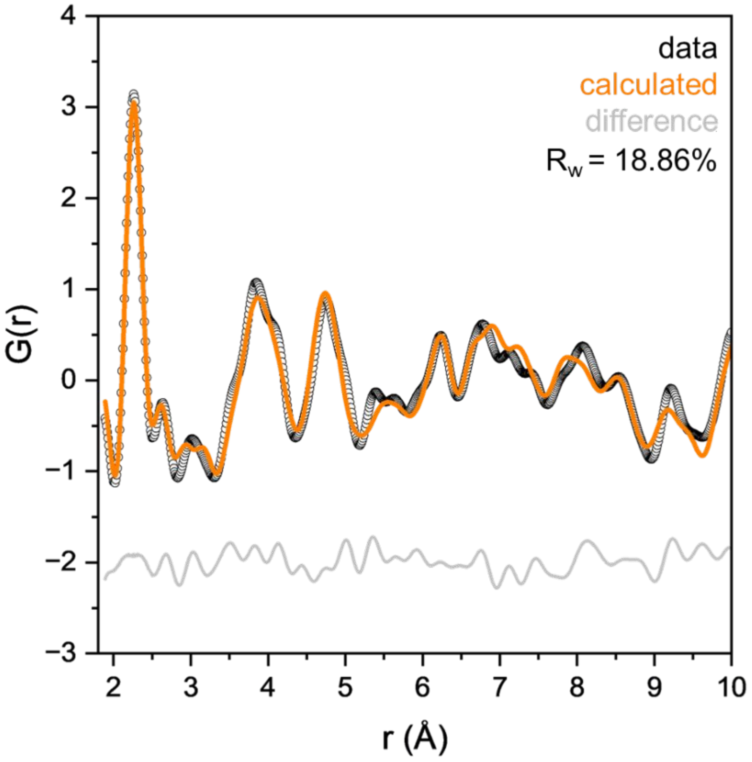

Figure S8: Fitting of the X-ray pair distribution function G(r) of $Li_4Ge_{0.7}Sb_{0.3}S_4$ shows reasonable agreement with the average structure determined from PXRD. Rietveld refinement detected the presence of another phase, $Li_{17}Sb_{13}S_{11}$ which is not included in this fit and is most likely responsible for the worse fit in this case. Data, calculated model, and difference curve are shown in black, orange and gray, respectively.

Electrochemical Impedance Spectroscopy Figures
Figure S9: Li_4GeS_4

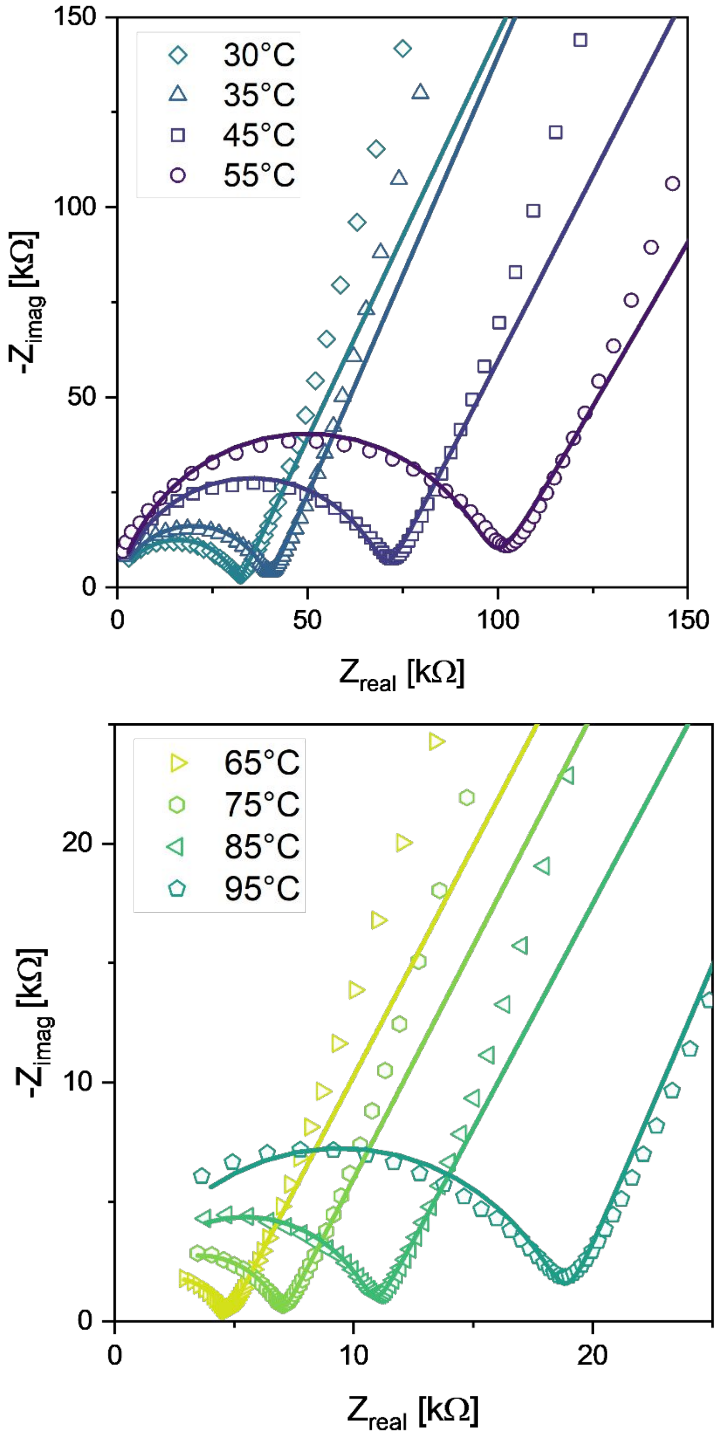

Figure S9: Selected Nyquist plots of the temperature dependent impedance spectroscopy for Li_4GeS_4. Data are shown as open polygons while the fit is shown as a line. The data were modeled with an $RQ + Q$ equivalent circuit.

Figure S10: $Li_{3.7}Ge_{0.7}P_{0.3}S_4$

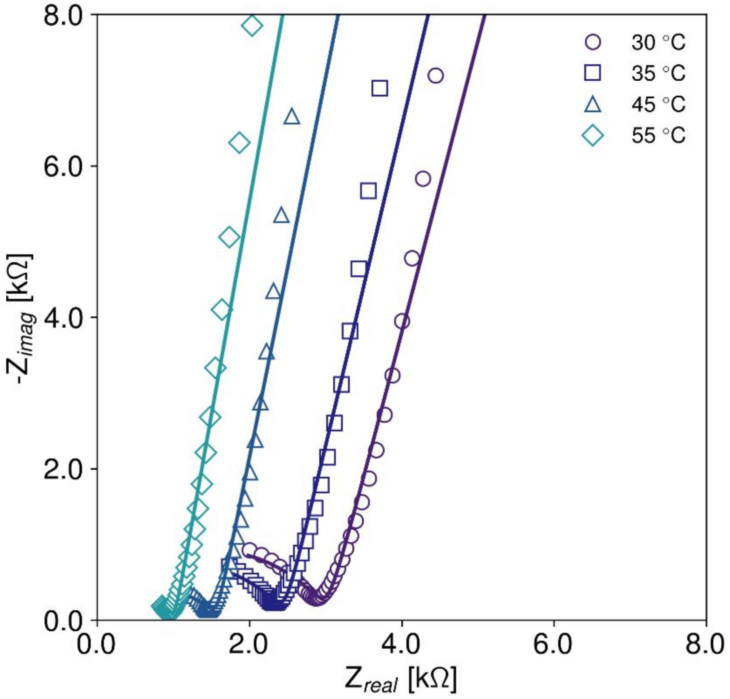

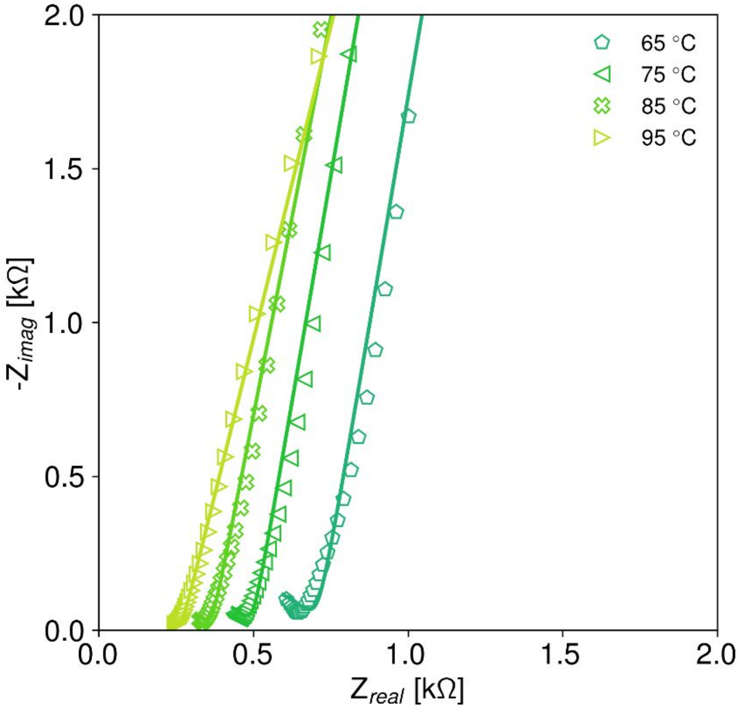

Figure S10: Selected Nyquist plots of the temperature dependent impedance spectroscopy for $Li_4Ge_{0.7}P_{0.3}S_4$. Data are shown as open polygons while the fit is shown as a line. The data were modeled with an *RQ* + *Q* equivalent circuit.

Figure S11: $Li_{3.7}Ge_{0.7}As_{0.3}S_4$

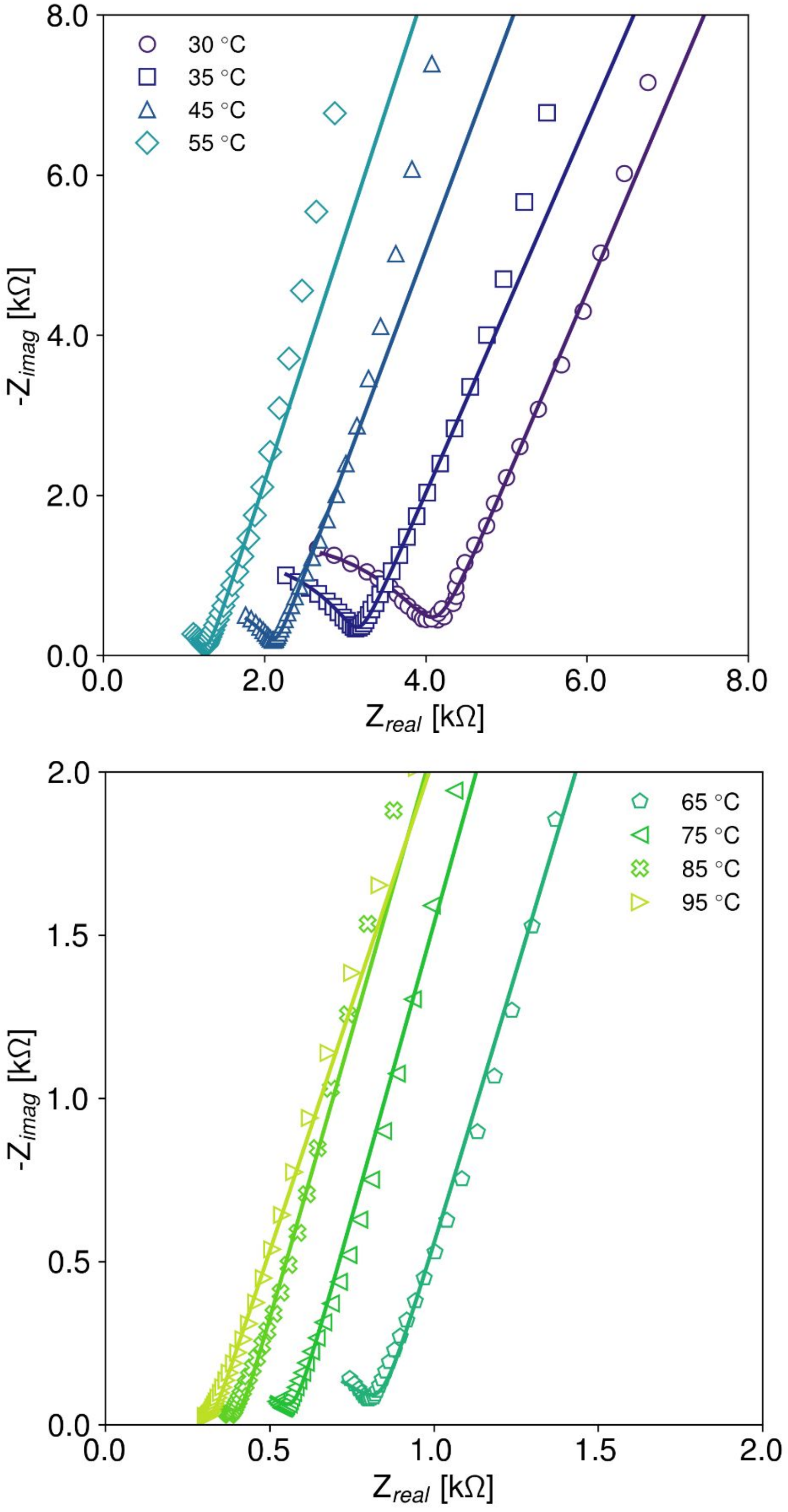

Figure S11: Selected Nyquist plots of the temperature dependent impedance spectroscopy for $Li_4Ge_{0.7}As_{0.3}S_4$. Data are shown as open polygons while the fit is shown as a line. The data were modeled with an *RQ* + *Q* equivalent circuit.

Figure S12: $Li_{3.7}Ge_{0.7}Sb_{0.3}S_4$

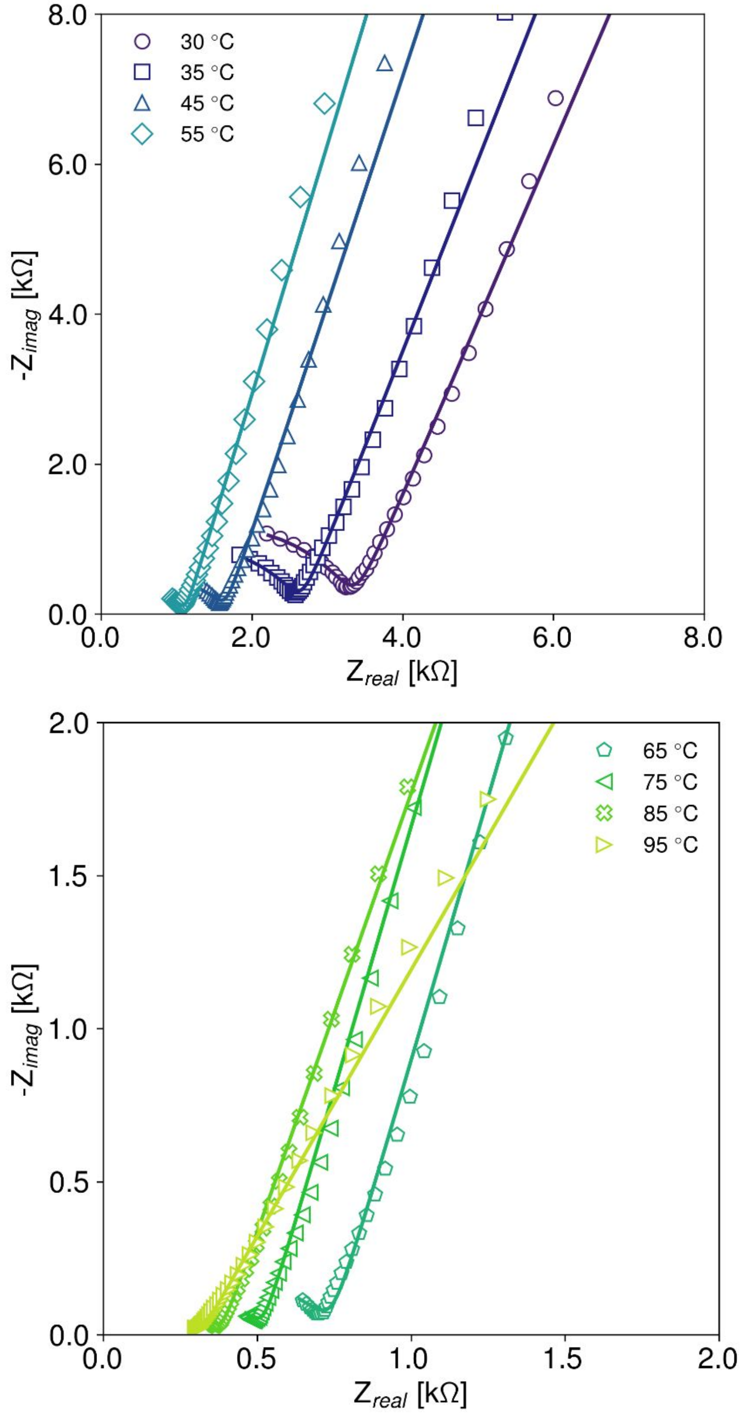

Figure S12: Selected Nyquist plots of the temperature dependent impedance spectroscopy for $Li_4Ge_{0.7}Sb_{0.3}S_4$. Data are shown as open polygons while the fit is shown as a line. The data were modeled with an $RQ + Q$ equivalent circuit.

Table S6: EIS fitting parameter Table

T (°C)	R1 (Ω)	σ (S·cm^{-1})	n1	Q1 (S/s^n)	C1 (F)	τ1 (Hz)	n2	Q2 (S/s^n)
$Li_{3.7}Ge_{0.7}P_{0.3}S_4$								
T (°C)	R1 (Ω)	σ (S·cm^{-1})	n1	Q1 (S/s^n)	C1 (F)	τ1 (Hz)	n2	Q2 (S/s^n)
30	3.01E+03	1.16E-04	0.72	1.56E-09	1.37E-11	3.87E+06	0.82	1.53E-07
35	2.28E+03	1.53E-04	0.8	4.73E-10	1.38E-11	5.06E+06	0.73	4.12E-07
45	1.42E+03	2.45E-04	0.8	5.02E-10	1.37E-11	8.19E+06	0.76	3.35E-07
55	9.28E+02	3.75E-04	0.79	6.44E-10	1.31E-11	1.31E+07	0.78	3.09E-07
65	6.46E+02	5.39E-04	0.75	1.18E-09	1.14E-11	2.15E+07	0.79	2.96E-07
75	4.60E+02	7.56E-04	0.67	4.71E-09	8.25E-12	4.19E+07	0.8	3.02E-07
85	3.41E+02	1.02E-03	0.6	1.75E-08	5.76E-12	8.11E+07	0.8	3.66E-07
95	2.54E+02	1.37E-03	0.6	1.93E-08	5.56E-12	1.13E+08	0.79	5.45E-07
$Li_{3.7}Ge_{0.7}As_{0.3}S_4$								
T (°C)	R1 (Ω)	σ (S·cm^{-1})	n1	Q1 (S/s^n)	C1 (F)	τ1 (Hz)	n2	Q2 (S/s^n)
30	3.96E+03	9.04E-05	0.79	3.92E-10	1.15E-11	3.50E+06	0.73	2.05E-07
35	3.17E+03	1.13E-04	0.79	4.33E-10	1.14E-11	4.42E+06	0.75	1.91E-07
45	1.93E+03	1.85E-04	0.82	2.78E-10	1.18E-11	6.97E+06	0.76	2.06E-07
55	1.24E+03	2.89E-04	0.82	3.38E-10	1.29E-11	9.95E+06	0.77	2.19E-07
65	8.10E+02	4.42E-04	0.74	1.37E-09	1.07E-11	1.84E+07	0.79	2.16E-07
75	5.79E+02	6.19E-04	0.6	1.41E-08	5.70E-12	4.82E+07	0.8	2.22E-07
85	4.12E+02	8.69E-04	0.6	1.74E-08	6.46E-12	5.98E+07	0.8	2.83E-07
95	2.95E+02	1.22E-03	0.6	1.99E-08	6.46E-12	8.36E+07	0.77	5.01E-07
$Li_{3.7}Ge_{0.7}Sb_{0.3}S_4$								
T (°C)	R1 (Ω)	σ (S·cm^{-1})	n1	Q1 (S/s^n)	C1 (F)	τ1 (Hz)	n2	Q2 (S/s^n)
30	3.25E+03	9.46E-05	0.79	4.67E-10	1.31E-11	3.74E+06	0.72	2.99E-07
35	2.49E+03	1.24E-04	0.79	5.06E-10	1.40E-11	4.56E+06	0.73	2.89E-07
45	1.58E+03	1.95E-04	0.8	4.86E-10	1.29E-11	7.80E+06	0.74	2.94E-07
55	1.06E+03	2.92E-04	0.78	6.90E-10	1.16E-11	1.30E+07	0.76	2.74E-07
65	7.23E+02	4.26E-04	0.67	4.70E-09	8.96E-12	2.46E+07	0.78	2.51E-07
75	5.11E+02	6.02E-04	0.6	1.70E-08	7.19E-12	4.33E+07	0.79	2.72E-07
85	3.95E+02	7.80E-04	0.6	1.66E-08	5.79E-12	6.97E+07	0.75	5.97E-07
95	2.93E+02	1.05E-03	0.6	1.12E-08	2.46E-12	2.21E+08	0.6	6.47E-06
Li_4GeS_4								
T (°C)	R1 (Ω)	σ (S·cm^{-1})	n1	Q1 (S/s^n)	C1 (F)	τ1 (Hz)	n2	Q2 (S/s^n)
30	9.79E+04	2.83E-06	0.87	6.77E-11	1.15E-11	1.41E+05	0.67	1.63E-07
35	6.97E+04	3.97E-06	0.87	6.99E-11	1.13E-11	2.02E+05	0.7	1.47E-07
45	3.94E+04	7.02E-06	0.87	7.61E-11	1.12E-11	3.60E+05	0.74	1.33E-07
55	3.16E+04	8.77E-06	0.85	1.09E-10	1.14E-11	4.41E+05	0.72	2.01E-07
65	1.87E+04	1.48E-05	0.83	1.46E-10	1.15E-11	7.41E+05	0.75	2.00E-07
75	1.07E+04	2.58E-05	0.86	1.02E-10	1.14E-11	1.30E+06	0.69	3.23E-07
85	6.94E+03	3.99E-05	0.85	1.37E-10	1.16E-11	1.98E+06	0.7	3.82E-07
95	4.65E+03	5.95E-05	0.83	1.87E-10	1.18E-11	2.91E+06	0.69	5.03E-07

Table S6: Table of values extracted from the fits of the Nyquist plots shown in Figures S4.1-S4.4. The equivalent circuit used for modeling consists of a ZARC element in series with a constant phase element (CPE). Sigma (σ) and C1 represent the ionic conductivity and capacitance attributed to bulk ion transport in all samples. Tau (τ1) represents the characteristic frequency of the ZARC element.

Table S7: Table of Activation Energies and Arrhenius Prefactor

Sample	Li_4GeS_4	$Li_{3.7}Ge_{0.7}P_{0.3}S_4$	$Li_{3.7}Ge_{0.7}As_{0.3}S_4$	$Li_{3.7}Ge_{0.7}Sb_{0.3}S_4$
Activation Energy (meV)	457	390	413	391
Ea Error (meV)	17	12	12	15
Intercept ln(σT) ($S\cdot K\cdot cm^{-1}$)	10.43832	11.64378	12.25138	11.95136
Prefactor ($S\cdot K\cdot cm^{-1}$)	3.41E+04	1.14E+05	2.09E+05	9.34E+04
Prefactor Error	2.27E+04	4.24E+03	7.15E+03	4.34E+03

Table S7: Table of activation energies and Arrhenius prefactors for all samples. The values were calculated from the linear regression of the Arrhenius plot of the temperature dependent impedance spectroscopy measurements.

Critical Current Density Figures
Figure S13: Chronopotentiometry Voltage/Current vs. Time

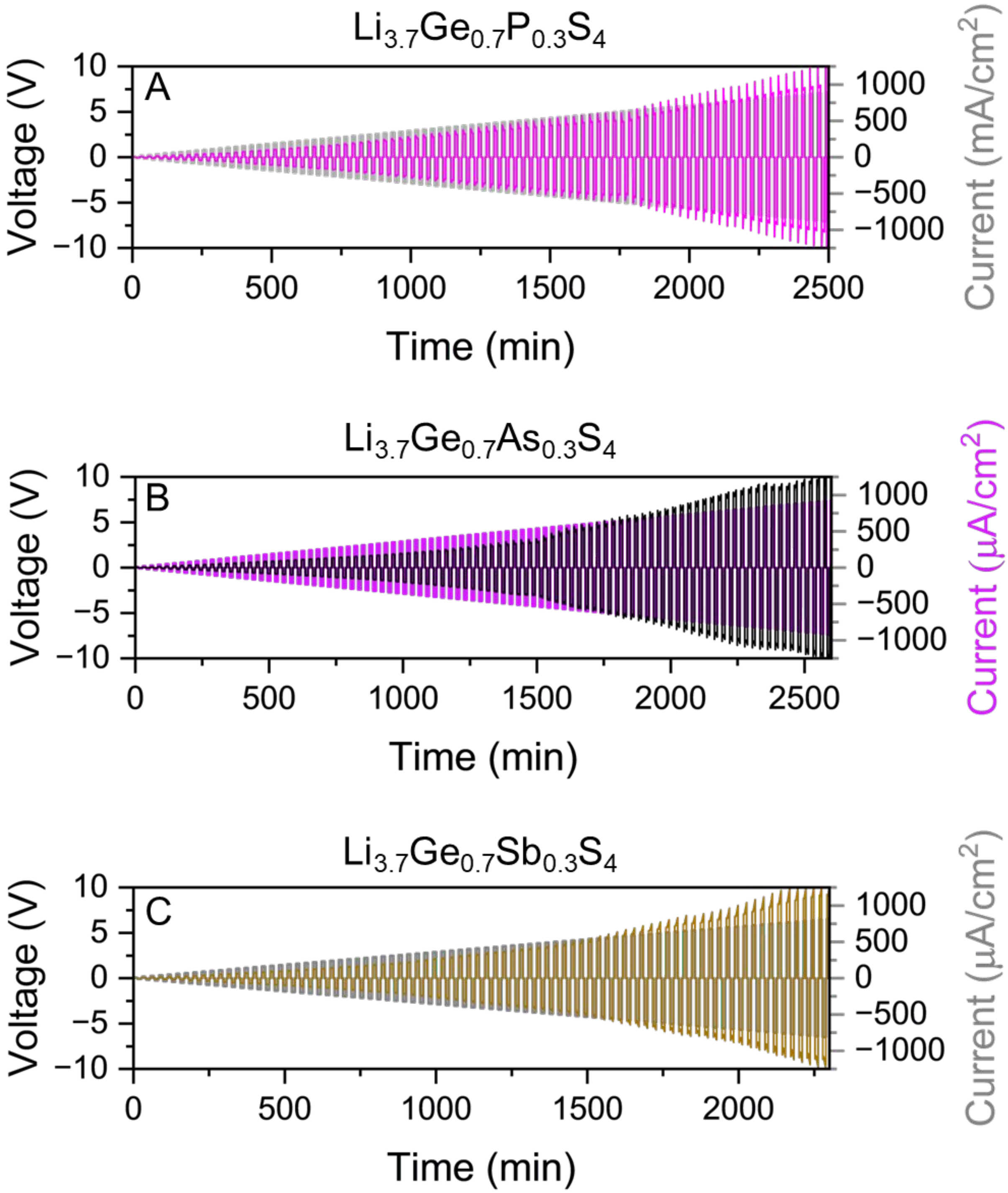

Figure S13: Voltage/current vs. time of the critical current density tests are plotted for $Li_{3.7}Ge_{0.7}Pn_{0.3}S_4$ (Pn = P(A), As(B), Sb(C)). Up to ~0.4 mA/cm^2 the voltage profiles are flat indicating a well conformed Li|SSE interface. At higher current densities, polarization of the voltage profiles can be observed.

Figure S14 Evolution of PEIS with Li symmetric cycling

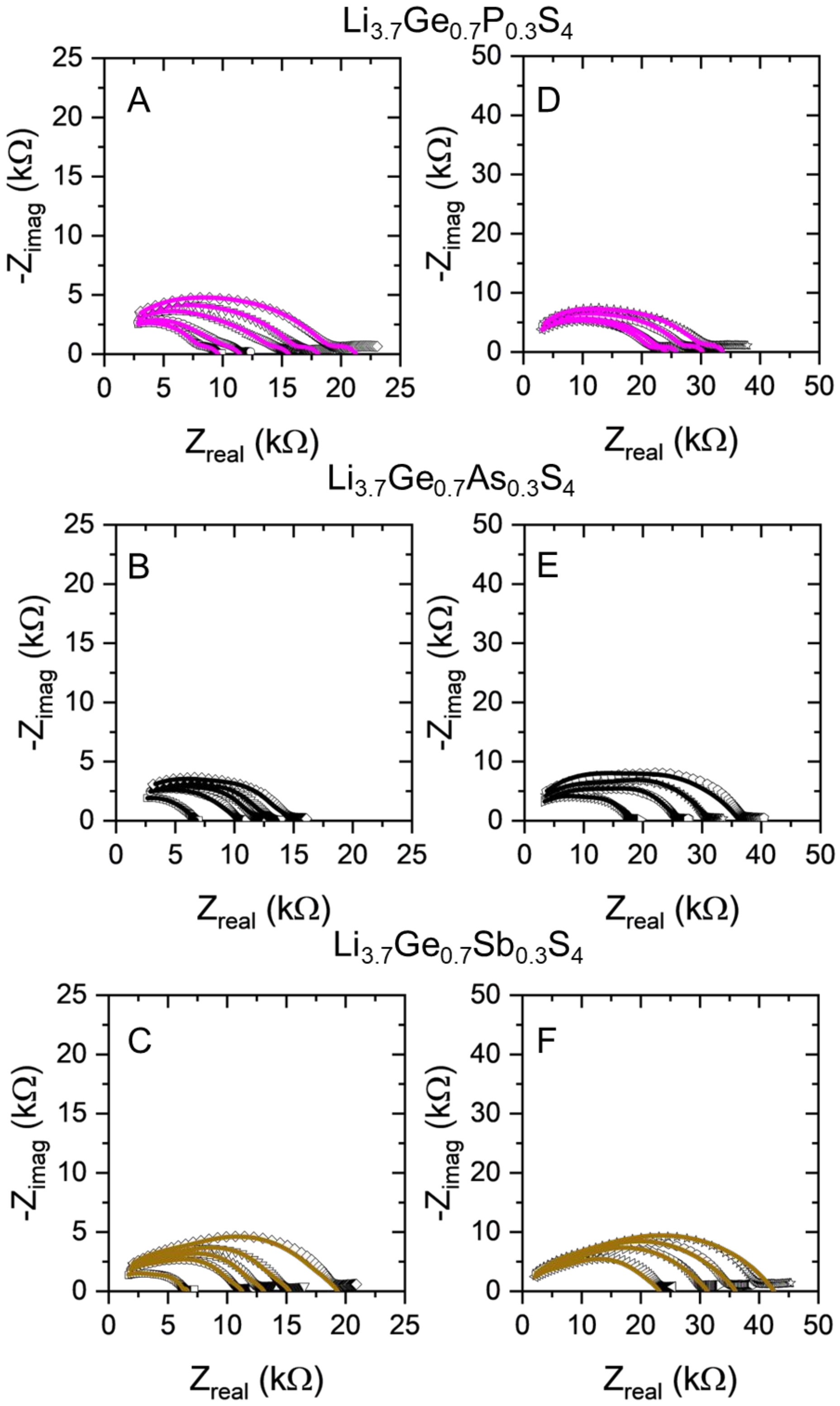

Figure S14: Impedance spectroscopy was collected throughout the critical current density tests. For each sample, the Nyquist plots of cycle 1, 10, 20, 30, 40, are plotted for P(A), As(B), Sb(C) and cycle 50, 60, 70, and 80 are plotted for P(D), As(E), Sb(F). All subsequent cycles resulted in an increase of the overall impedance of the cell. Data are plotted in open polygons while fits are plotted in solid lines. The equivalent circuit used for modelling consisted of 3 ZARC elements in

Figure S15 Equivalent Circuit for PEIS of CCD test

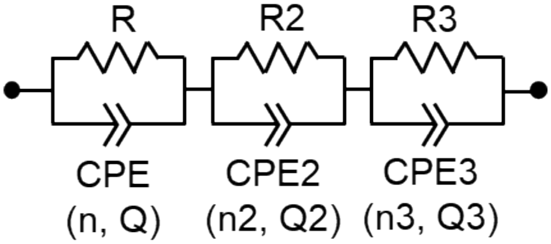

Figure S15: The equivalent circuit used for modelling the impedance data collected throughout the critical current density tests. The 3 ZARC elements correspond to the bulk, interphase, and interfacial transport. The majority of the increase in impedance is thought to be due to the growth of the solid electrolyte interphase ultimately driven by the reactivity between the SSE and Li metal.

Table S8: Table of extracted values from evolution of PEIS fits

$Li_{3.7}Ge_{0.7}P_{0.3}S_4$													
Cycle#	R (Ω)	n	Q	R2 (Ω)	n2	Q2	R3 (Ω)	n3	Q3	σ (S·cm^{-1})	Cap1 (F)	Cap2 (F)	Cap3 (F)
1	4002.553	0.835188	2.64E-10	3576.299	0.811054	1.30E-09	2056.716	0.6	7.46E-07	6.52E-05	2.17E-11	2.69E-10	3.10E-07
10	7219.088	0.762679	8.08E-10	2846.243	0.6	1.21E-07	1397.2	0.6	2.53E-06	3.61E-05	2.62E-11	9.10E-11	8.52E-07
20	7499.999	0.741155	1.15E-09	2922.359	0.6	2.00E-07	835.582	0.6	1.17E-05	3.48E-05	3.16E-11	4.30E-11	4.48E-06
30	4555.382	0.856152	3.14E-10	11712.38	0.6	2.08E-08	1803.667	0.6	1.11E-05	5.73E-05	3.51E-11	3.96E-11	6.11E-06
40	4000.031	0.887616	2.30E-10	15090.15	0.6	1.68E-08	2202.095	0.6	1.38E-05	6.52E-05	2.61E-11	9.35E-11	8.88E-07
50	4150.422	0.912028	1.54E-10	17402.35	0.6	1.57E-08	2569.242	0.6	1.27E-05	6.29E-05	1.95E-11	1.40E-09	5.33E-07
60	5471.254	0.877663	2.14E-10	17532.47	0.6	1.74E-08	2843.451	0.6	1.14E-05	4.77E-05	2.87E-11	5.09E-11	2.51E-06
70	7500	0.87608	1.77E-10	19538.63	0.6	1.85E-08	3309.03	0.6	9.14E-06	3.48E-05	1.95E-11	1.40E-09	5.33E-07
80	7500	0.924806	8.22E-11	22544.24	0.6	1.56E-08	3715.486	0.6	6.47E-06	3.48E-05	2.58E-11	8.60E-11	7.41E-07
$Li_{3.7}Ge_{0.7}As_{0.3}S_4$													
Cycle#	R (Ω)	n	Q	R2 (Ω)	n2	Q2	R3 (Ω)	n3	Q3	σ (S·cm^{-1})	Cap1 (F)	Cap2 (F)	Cap3 (F)
1	4000	0.755999	1.10E-09	2693.516	0.6	4.74E-08	115.745	1	1.00E-06	7.47E-05	2.81E-10	1.37E-08	1.53E-11
10	4000	0.807045	2.00E-09	2049.613	0.6	2.86E-07	4188.745	0.830064	2.57E-10	7.47E-05	4.70E-11	1.13E-08	2.52E-11
20	4000	0.853277	2.00E-09	1533.345	0.6	1.00E-06	6569.836	0.788995	4.55E-10	7.47E-05	1.21E-10	2.00E-09	1.54E-11
30	4000	0.862726	2.00E-09	1690.536	0.6	1.00E-06	7483.107	0.775292	5.63E-10	7.47E-05	3.09E-10	1.42E-08	1.56E-11
40	4000	0.877719	2.00E-09	1897.796	0.6	1.00E-06	8952.384	0.741729	9.08E-10	7.47E-05	8.47E-11	1.18E-10	2.18E-11
50	4000.094	0.932118	1.74E-09	2265.692	0.6	1.00E-06	12190.17	0.688021	2.20E-09	7.47E-05	6.72E-10	1.68E-08	2.03E-11
60	5981.819	0.927099	2.00E-09	2808.777	0.6	1.00E-06	17602.05	0.617747	5.57E-09	4.99E-05	8.20E-10	4.75E-10	1.92E-11
70	4000	0.954791	2.00E-09	14735.56	0.6	4.73E-08	12533.08	0.749612	8.88E-10	7.47E-05	9.04E-11	1.07E-10	2.36E-11
80	4000	0.934414	2.00E-09	16474.62	0.6	4.53E-08	15839.2	0.780644	4.82E-10	7.47E-05	1.18E-09	3.35E-10	1.95E-11
$Li_{3.7}Ge_{0.7}Sb_{0.3}S_4$													
Cycle#	R (Ω)	n	Q	R2 (Ω)	n2	Q2	R3 (Ω)	n3	Q3	σ (S·cm^{-1})	Cap1 (F)	Cap2 (F)	Cap3 (F)
1	4000	0.735114	2.00E-09	2325.256	0.82572	8.79E-09	327.6558	1	1.00E-06	5.24E-05	2.97E-11	4.31E-10	1.70E-08
10	5366.369	0.73116	2.00E-09	4072.015	0.898775	1.78E-09	1839.817	0.6	1.00E-06	3.90E-05	4.41E-11	1.16E-09	3.02E-10
20	6098.577	0.728822	2.00E-09	4907.222	0.896627	1.69E-09	2097.062	0.6	1.00E-06	3.43E-05	2.85E-11	6.56E-10	1.20E-08
30	6669.144	0.727057	2.00E-09	6236.472	0.874043	2.06E-09	2362.496	0.6	1.00E-06	3.14E-05	2.96E-11	4.20E-10	1.75E-08
40	7117.296	0.727246	2.00E-09	9409.564	0.812138	3.96E-09	2805.568	0.6	1.00E-06	2.94E-05	4.41E-11	1.16E-09	3.02E-10
50	7351.283	0.728487	2.00E-09	12516.68	0.775445	5.90E-09	3292.234	0.6	1.00E-06	2.85E-05	2.97E-11	4.55E-10	1.55E-08
60	7500	0.74106	1.66E-09	19933.69	0.719942	9.28E-09	3813.724	0.6	1.00E-06	2.79E-05	3.22E-11	3.40E-10	2.38E-08
70	7500	0.763599	1.14E-09	24681.96	0.692896	1.13E-08	3851.299	0.6	1.00E-06	2.79E-05	2.96E-11	4.06E-10	1.77E-08
80	7500	0.744411	2.00E-09	50	0.999997	1.16E-09	34981.81	0.6	2.95E-08	2.79E-05	3.08E-11	3.78E-10	2.03E-08

Table S8: Table of the extracted parameters from the fits of the impedance data shown in Figure S14 and modeled with the equivalent circuit shown in Figure S15.

Virtual Electrode XPS figures

Figure S16: $Li_{3.7}Ge_{0.7}P_{0.3}S_4$ VE-XPS

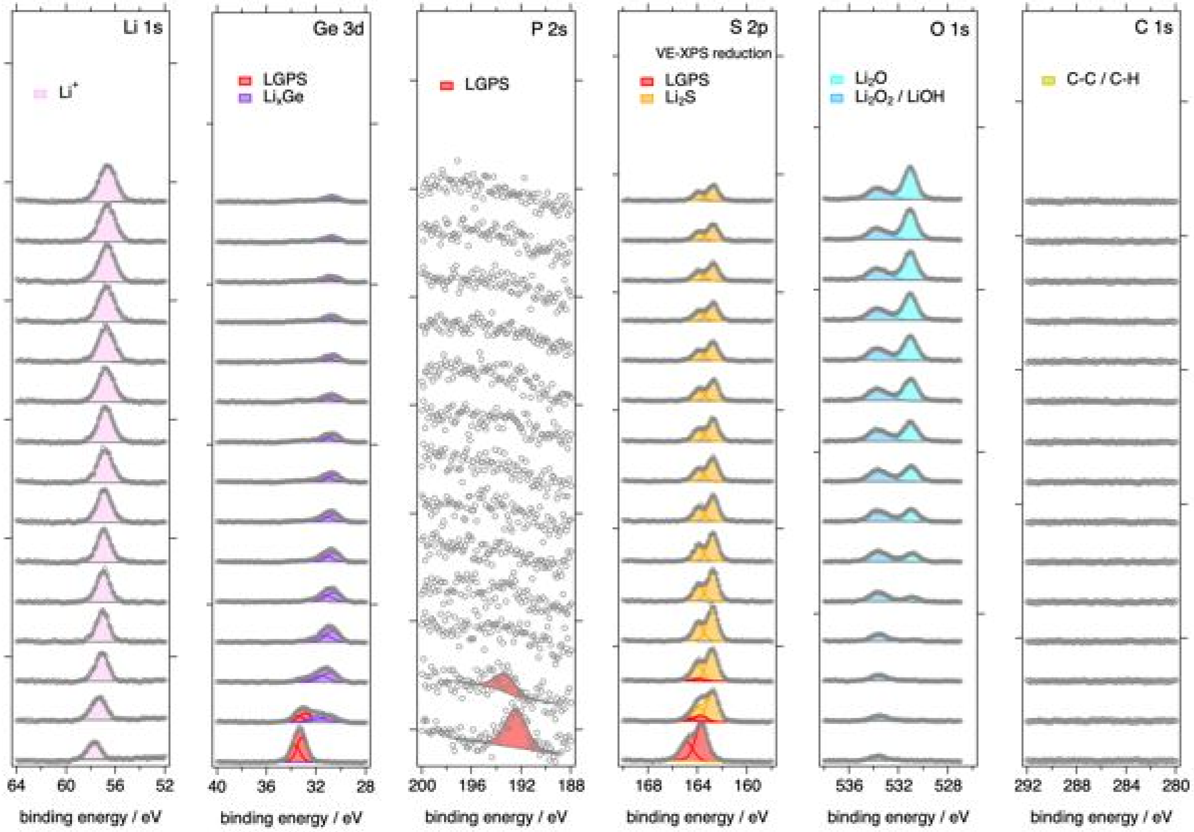

Figure S16: Evolution of the XPS spectra collected throughout the virtual electrode experiment of $Li_{3.7}Ge_{0.7}P_{0.3}S_4$. The surface is dominated by formation of Li_2S as well as Li_2O and Li_2O_2/Li_2OH. Li^0 plating was not observed.

Figure S17: $Li_{3.7}Ge_{0.7}As_{0.3}S_4$ VE-XPS

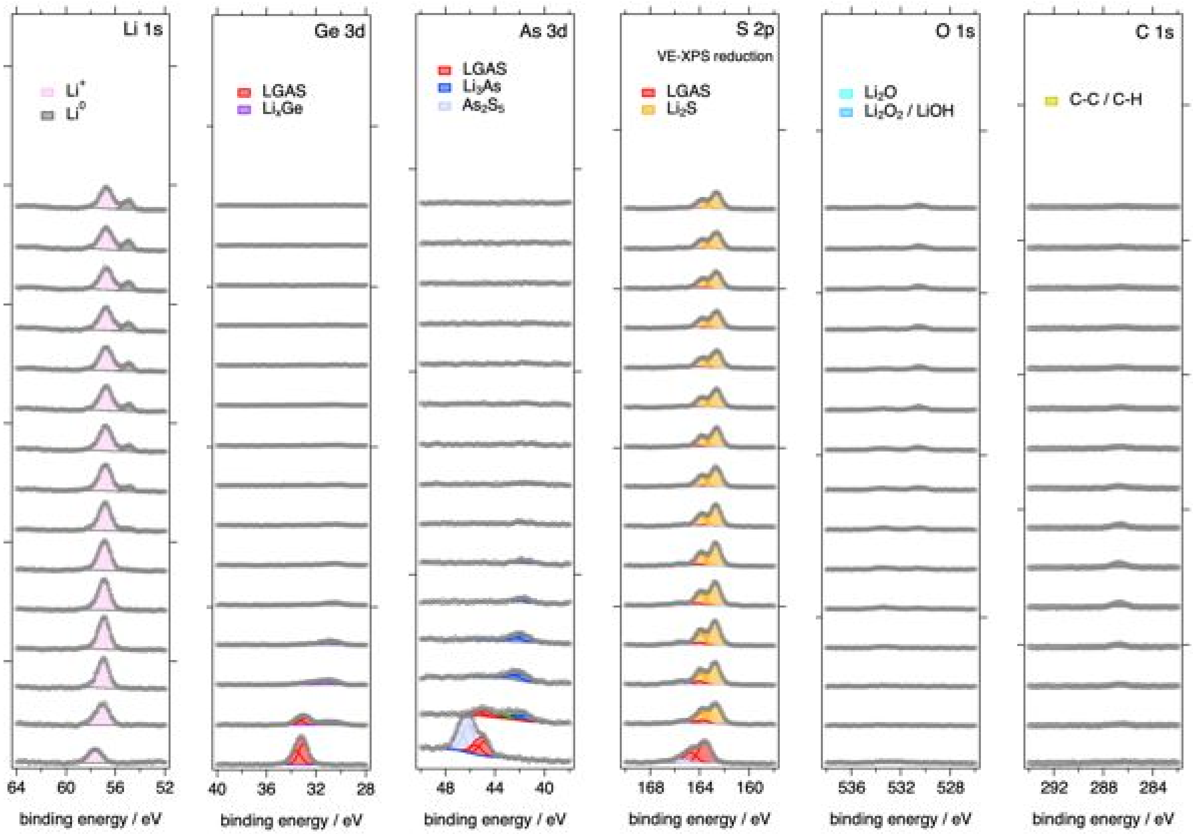

Figure S17: Evolution of the XPS spectra collected throughout the virtual electrode experiment of $Li_{3.7}Ge_{0.7}As_{0.3}S_4$. Li metal can be observed at the surface suggesting passivating of the SSE surface. Only a small amount of Li_2O is observed in this case.

Figure S18: $Li_{3.7}Ge_{0.7}Sb_{0.3}S_4$ VE-XPS

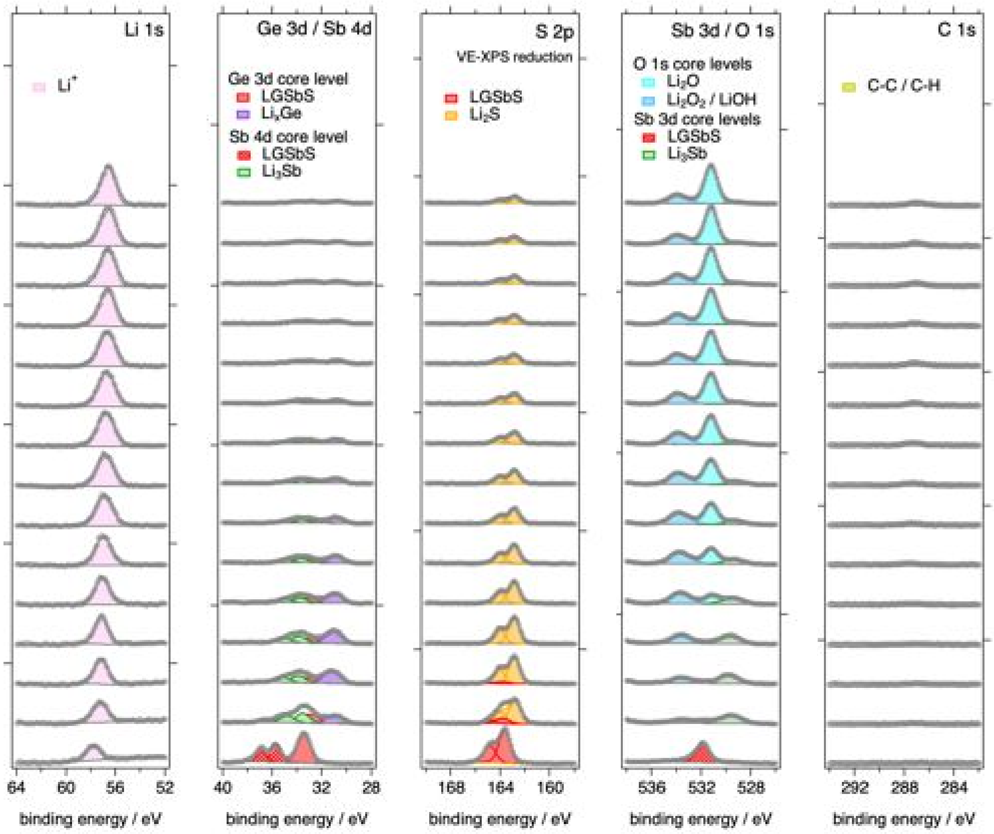

Figure S18: Evolution of the XPS spectra collected throughout the virtual electrode experiment of $Li_{3.7}Ge_{0.7}Sb_{0.3}S_4$. The surface is dominated by formation of Li_2S as well as Li_2O and Li_2O_2/Li_2OH. Li^0 plating was not observed.

Figure S19: Phase Composition vs. XPS cycle number

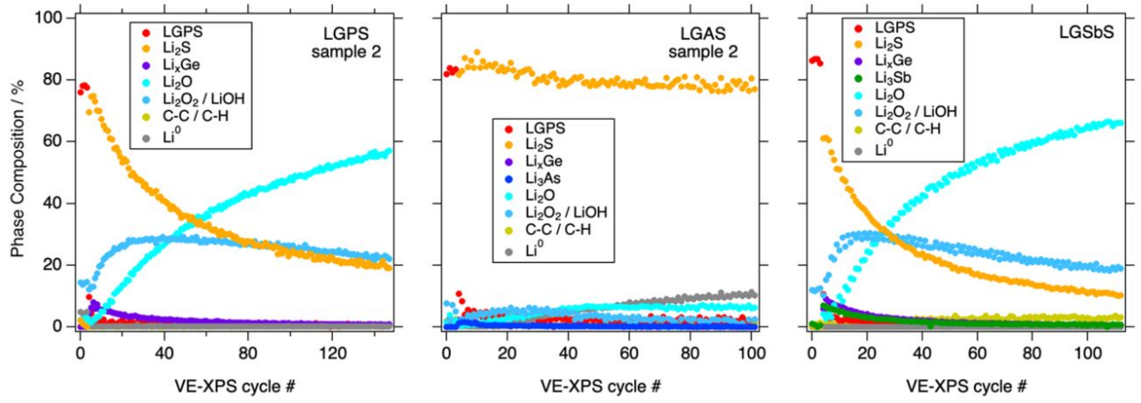

Figure S19: Phase composition vs. XPS cycle throughout the virtual electrode experiment shows that in the case of $Li_{3.7}Ge_{0.7}P_{0.3}S_4$ and $Li_{3.7}Ge_{0.7}Sb_{0.3}S_4$ the surface is dominated by Li_2S and oxide/hydroxide compounds. However, in the case of $Li_{3.7}Ge_{0.7}As_{0.3}S_4$ the surface is dominated by Li_2S and Li^0 plating can be observed. In all cases the degradation of the solid electrolyte is apparent.
